# Outbreak report of polymyxin-carbapenem-resistant *Klebsiella pneumoniae* causing untreatable infections evidenced by synergy tests and bacterial genomes

**DOI:** 10.1038/s41598-023-31901-4

**Published:** 2023-04-17

**Authors:** Marisa Zenaide Ribeiro Gomes, Elisangela Martins de Lima, Caio Augusto Martins Aires, Polyana Silva Pereira, Juwon Yim, Fernando Henrique Silva, Caio Augusto Santos Rodrigues, Thamirys Rachel Tavares e Oliveira, Priscila Pinho da Silva, Cristiane Monteiro Eller, Claudio Marcos Rocha de Souza, Michael J. Rybak, Rodolpho Mattos Albano, Antonio Basílio de Miranda, Edson Machado, Marcos Catanho, Nucleus of Hospital Research (NPH) study collaborators, Vitoria Pinson Ruggi Dutra, Luciana Sênos de Mello, João Pedro Silva Tonhá, Murillo Marçal Castro, Amanda Aparecida da Silva Machado, Maxuel Cassiano da Silva, Yann Rodrigues Mathuiy, Thaisa Medeiros Tozo

**Affiliations:** 1 Present Address: Laboratório de Genética Molecular de Microrganismos, Instituto Oswaldo Cruz, Fundação Oswaldo Cruz, Rio de Janeiro, Brazil; 2 Hospital Federal Servidores do Estado, Ministry of Health, Rio de Janeiro, Brazil; 3 Laboratório de Pesquisa em Infecção Hospitalar, Instituto Oswaldo Cruz, Fundação Oswaldo Cruz, Rio de Janeiro, RJ Brazil; 4 Anti-Infective Research Laboratory, Eugene Applebaum College of Pharmacy and Health Sciences, Department of Medicine, Division of Infectious Diseases, School of Medicine, Wayne State University, Detroit, MI USA; 5 Departamento de Bioquímica, IBRAG, Universidade do Estado do Rio de Janeiro,, Rio de Janeiro, Brazil; 6 Hospital Infection Control Committee, Hospital Universitário Pedro Ernesto, Universidade do Estado do Rio de Janeiro, Rio de Janeiro, Brazil; 7 Present Address: Departamento de Ciência da Saúde, Universidade Federal Rural do Semi-Árido (UFERSA), Mossoró, Rio Grande do Norte Brazil; 8 Present Address: Laboratório de Biologia Molecular Aplicada a Micobactérias, Instituto Oswaldo Cruz, Fundação Oswaldo Cruz, Rio de Janeiro, Brazil

**Keywords:** Computational biology and bioinformatics, Microbiology, Molecular biology, Health care, Medical research

## Abstract

Polymyxin-carbapenem-resistant *Klebsiella pneumoniae* (PCR-Kp) with pan (PDR)- or extensively drug-resistant phenotypes has been increasingly described worldwide. Here, we report a PCR-Kp outbreak causing untreatable infections descriptively correlated with bacterial genomes. Hospital-wide surveillance of PCR-Kp was initiated in December-2014, after the first detection of a *K. pneumoniae* phenotype initially classified as PDR, recovered from close spatiotemporal cases of a sentinel hospital in Rio de Janeiro. Whole-genome sequencing of clinical PCR-Kp was performed to investigate similarities and dissimilarities in phylogeny, resistance and virulence genes, plasmid structures and genetic polymorphisms. A target phenotypic profile was detected in 10% (12/117) of the tested *K. pneumoniae* complex bacteria recovered from patients (8.5%, 8/94) who had epidemiological links and were involved in intractable infections and death, with combined therapeutic drugs failing to meet synergy. Two resistant bacterial clades belong to the same transmission cluster (ST437) or might have different sources (ST11). The severity of infection was likely related to patients’ comorbidities, lack of antimicrobial therapy and predicted bacterial genes related to high resistance, survival, and proliferation. This report contributes to the actual knowledge about the natural history of PCR-Kp infection, while reporting from a time when there were no licensed drugs in the world to treat some of these infections. More studies comparing clinical findings with bacterial genetic markers during clonal spread are needed.

## Introduction

At present, the dissemination of polymyxin-carbapenem-resistant *Klebsiella pneumoniae* (PCR-Kp) precludes treatment, posing a greater risk to human health, especially in low- and middle-income countries with limited access to newly developed drugs^1^. The most prevalent mechanism of carbapenem resistance is the production of carbapenemase, in which the enzyme hydrolyzes not only carbapenems but also several other beta-lactam antibiotics^2^. Carbapenemase-encoding plasmids are frequently vectors of resistance determinants for other antimicrobial classes, such as aminoglycosides and fluoroquinolones^3^. Resistance to polymyxins comprises chromosomal mutations or acquisition of the *mcr*-1 gene^4–6^, leading to extensive (XDR)- and pan (PDR)-drug resistant phenotypes among *K. pneumoniae* isolates.

Lethal outbreaks caused by PCR-Kp emerged as multilocus sequence type (MLST) 258 in the USA in 2009^7^, ST437 in Brazil in 2014 and 2015^8^, ST147 and ST101 in Greece in 2014 to 2016^9^, ST11 in Brazil in 2015 and 2016^10^ and ST307 in Germany in 2019^11^. ST258, ST11, ST437 and ST101 belong to the world’s most common clonal complex 258 (CC258), while the other STs have been growing in recognition^9,11^.

Factors associated with hypervirulence in PCR-Kp have recently been described in Germany^11^, India^12^ and China^13^, in which characteristics related to hypermucoviscosity and enhanced iron acquisition were detected in the strains of the ST307 outbreak^11^, ST5235 case series^12^ and evolved ST11 strains^13^. The confluence of hypervirulence features in carbapenemase-producing *K. pneumoniae* strains arose in the last decade in intensive care patients causing deadly outbreaks in Asia, associated with the acquisition of a large virulence plasmid or integrative conjugal elements (ICEs)^14^. On the other hand, hypervirulent *K. pneumoniae* (hvKp) strains have gained carbapenemase-encoding genes by acquiring resistance plasmids^15^. The coexistence of hyperresistance and hypervirulence in *K. pneumoniae* represents a continuous tendency due to the pathogen’s ability to adapt to environmental conditions and exchange genetic material^11,14,15^.

In this study, we report a lethal outbreak caused by *K. pneumoniae* with concomitant resistance to carbapenem and polymyxin, corroborated by antimicrobial synergy testing, in a tertiary public hospital in Rio de Janeiro^8^, in which all *K. pneumoniae* complex phenotypes were prospectively followed and classified according to published definitions^16^. Phylogenetic analysis and a detailed investigation of genetic similarities and dissimilarities in resistance and virulence genes, plasmid structures and polymorphisms of the clinical PCR-Kp (target resistance) were analyzed also considering clinical and epidemiological characteristics of infected patients, and the spatial monitoring methodology^17^. This approach aimed to improve the understanding of infectious processes and outbreaks caused by PCR-Kp.

## Results

### Emergence of PCR-Kp

**Figure 1:**
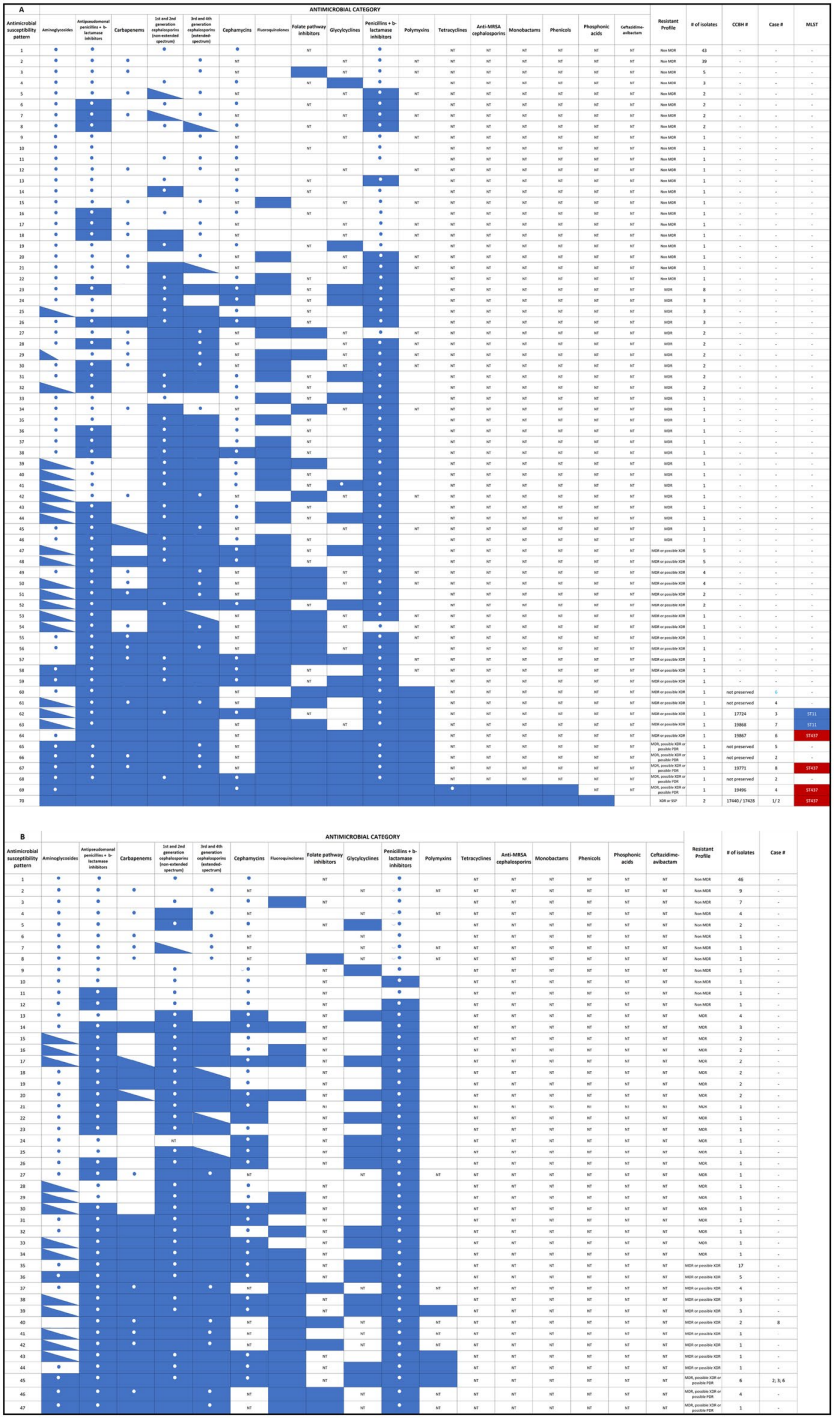

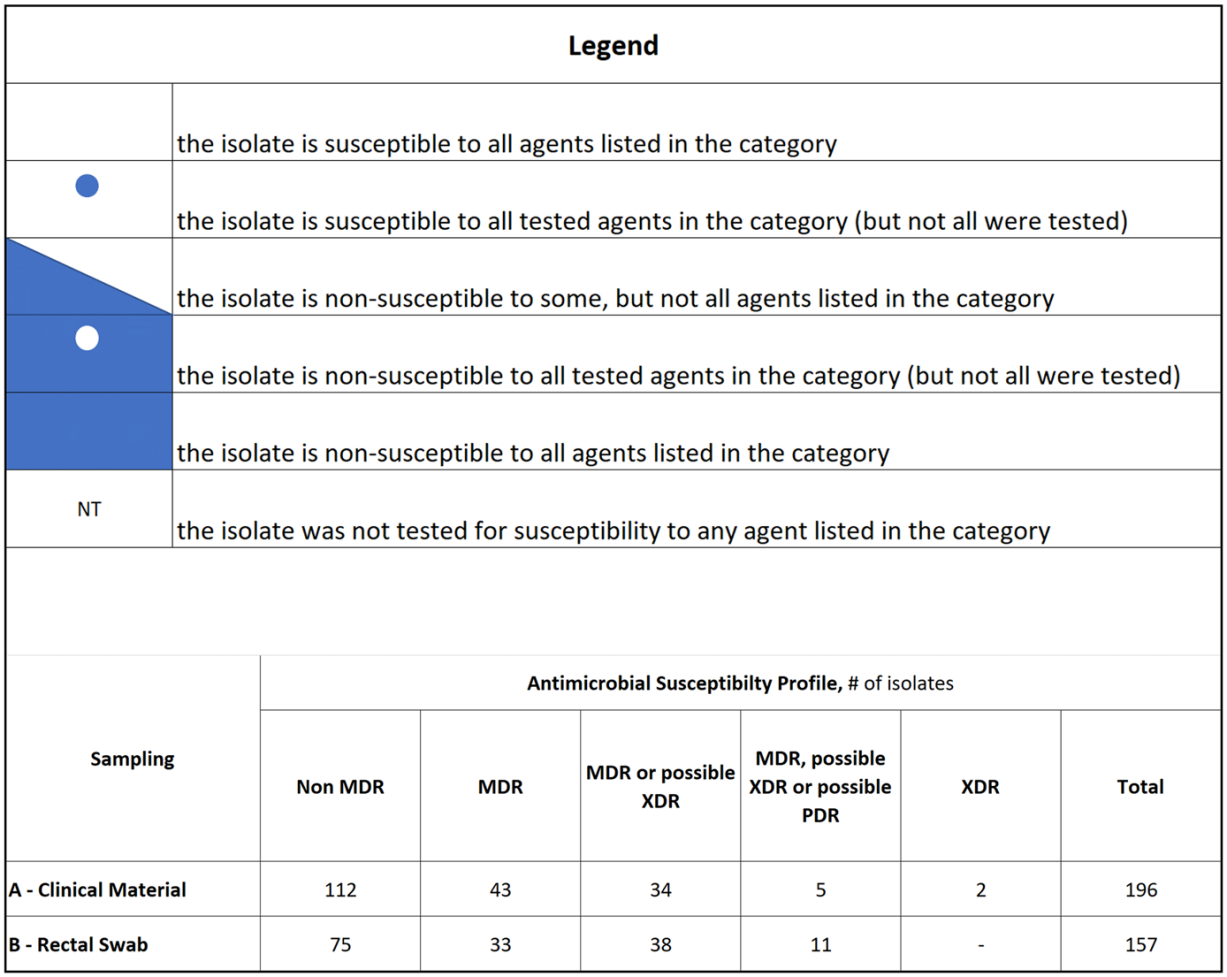
Antimicrobial susceptibility profile of *K. pneumoniae* complex isolates from clinical (**A**) and surveillance samples (**B**), according to Magiorakos et al*.* (2012) definitions^16^, December 2014 to August 2015, federal tertiary hospital, Rio de Janeiro, Brazil. Target clinical polymyxin-carbapenem-resistant *K. pneumoniae* strains of distinct MLST recovered from the studied cases are presented: ST437 strains highlighted in red; ST11 highlighted in blue. *MDR* multidrug resistant, *MLST* multilocus sequence typing, *MRSA* methicillin-resistant *Staphylococcus aureus*, *SSP* single susceptible profile, *XDR* extensively-drug resistant, *PDR* pandrug resistant.

The distribution of the antimicrobial susceptibility profile of the *K. pneumoniae* complex among a total of 353 nonrepetitive isolates from 196 clinical samples and 157 surveillance rectal swabs from 258 hospitalized patients is shown in Fig. 1. Supplementary Algorithm 1 shows *K. pneumoniae* complex isolates investigated according to the type of sample (clinical or surveillance) and resistance profile to carbapenems and polymyxins.

Carbapenem-resistant (meropenem, imipenem or ertapenem-intermediate/resistant) *K. pneumoniae* (CR-Kp) complex isolates were detected in 41% (64/157) of rectal swabs. In contrast, 93 (93/157, 59%) non-CR extended spectrum beta-lactamase (ESBL)-positive *K. pneumoniae* complex isolates comprised the remaining surveillance rectal swabs. Possible-PDR (n = 11) or possible-XDR (n = 38) patterns, according to the mentioned published definitions, were found in 77% (49/64) of CR-Kp complex strains from rectal swabs. Target concomitant resistance (CR-Kp complex isolates screened positive for resistance to polymyxins) was detected in 9% (11/128) of the swabs tested for any carbapenem and polymyxin through the Vitek-2 system (Biomérieux). These isolates corresponded to 17% (11/64) of CR-Kp complex recovered from surveillance rectal swabs. MICs for polymyxins and carbapenems were greater than or equal to 16 µg/ml in 82% (9/11) and 100% (11/11) of isolates, respectively, and were routinely retrieved from patients admitted to the medical-surgical intensive-care unit (MS-ICU) (n = 10) or in a surgical ward (n = 1), between January and April 2015 (n = 10) and in August 2015 (n = 1). None of the rectal swab isolates were preserved for additional tests (Supplementary Algorithm 1).

Among 196 clinical *K. pneumoniae* complex detected in 167 patients, 21% (41/196) of isolates had: (1) a single susceptible profile to ceftazidime-avibactam (CZA) confirmed later (n = 2 index strains) and a possible-PDR profile (n = 2 strains) recovered from the index cases during hospitalization in the infectious diseases ICU (n = 1 strain) and MS-ICU (n = 3 strains); and (2) possible-PDR (n = 3 strains) and possible-XDR (n = 34 strains) patterns found in isolates from other patients in the MS-ICU (n = 16 patients) and in the adult medical (n = 13) and surgical (n = 9) wards (Fig. 1). These strains were isolated from blood (21%, 12/58), respiratory secretions (46%, 6/13), urine (24%, 20/83) and other clinical samples (7%, 3/42). A high carbapenem minimum inhibitory concentration (MIC) ≥ 16 µg/ml was found in 94% (29/31) of all CR-Kp complex isolates detected*.* Phenotypic screening for carbapenemase production yielded positive results with boronic acid plus meropenem in 96% (24/25) of the tested CR-Kp complex strains. Screening for polymyxin/colistin resistance with the Vitek-2 system (MIC > 2 mg/L) was positive in 10% (12/117) of the isolates tested (56 isolates from blood, 13 from respiratory secretions, 10 from urine and 38 from other materials) with MIC values ≥ 16 mg/L in 82% (9/11) of strains (Fig. 1 and Supplementary Table 2). In total, we found target isolates (clinical CR-Kp complex isolates screened positive for resistance to polymyxins) in 40% (12/30) of CR-Kp strains screened for polymyxin resistance in eight patients (Supplementary Algorithm 1). Only seven target strains (7/12, 58%) recovered from clinical samples of seven (7/8, 88%) patients were preserved and had their genome analyzed.

**Figure 2:**
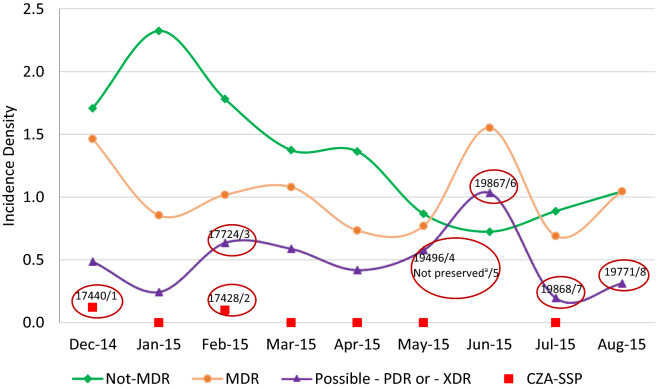
Incidence density of *K. pneumoniae* complex phenotypes detected in clinical samples/1000 patient-days, hospital-wide surveillance (n = 196 isolates; median of 22 isolates per month, range 17–26). The temporal occurrences of cases with polymyxin/carbapenem-resistant *K. pneumoniae* strains are represented with red circles (CCBH #/case #) over their corresponding phenotype curves. Case number in order of strain detection. Superscript a: not preserved *K. pneumoniae* complex isolate of case 5, that displayed carbapenem resistance and had positive screening for polymyxin resistance*.* *CZA-SSP* ceftazidime-avibactam single susceptible profile, *MDR* multidrug resistant, *XDR* extensively drug resistant, *PDR* pandrug resistant.

Figure 2 shows the monthly incidence density of all *K. pneumoniae* complex phenotypes and the temporal occurrences of laboratory-confirmed PCR-Kp strains (n = 7) detected in preserved clinical samples. Although CCBH17440 (case 1) and CCBH17428 (case 2) were the first noticed clinical *K. pneumoniae* strains with concomitant resistance to carbapenems and polymyxins, and initially classified as a possible PDR phenotype*,* a retrospective investigation confirmed this resistance profile screened in blood and secretion samples from a patient admitted to the MS-ICU 11 months earlier.

**Figure 3:**
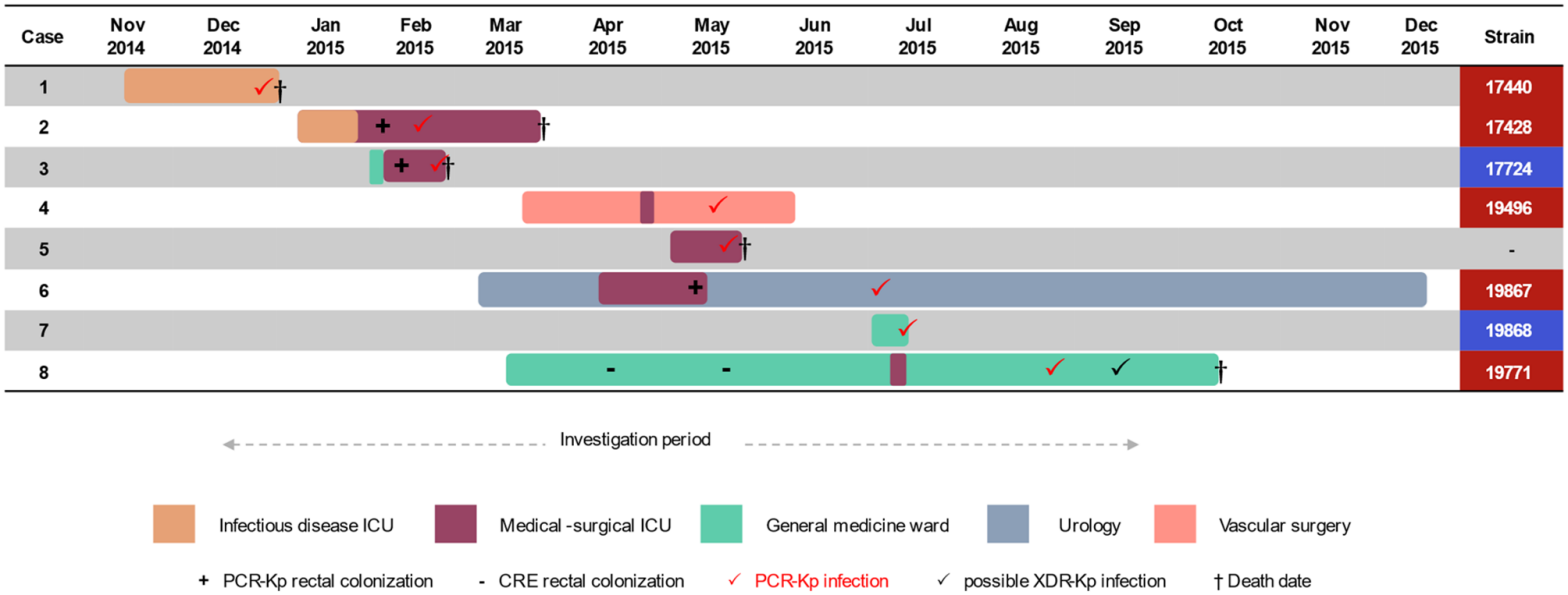
Timeline of infection. Gantt chart representing the unit and period of hospitalization of patients (cases 1–4 and 6–8) infected with polymyxin-carbapenem-resistant *K. pneumoniae* (PCR-Kp) of distinct MLST (ST437 strains, highlighted in red; ST11, in blue), during the 9 months of clinical sample surveillance from December 2014 to August 2015. Kp complex isolate screened as PCR profile from case 5 was not preserved for additional tests. Case number in order of strain detection. *CRE* carbapenem-resistant *Enterobacteriaceae*, *ICU* intensive care unit, *MLST* multilocus sequence typing; XDR, extensively drug-resistant.

**Figure 4:**
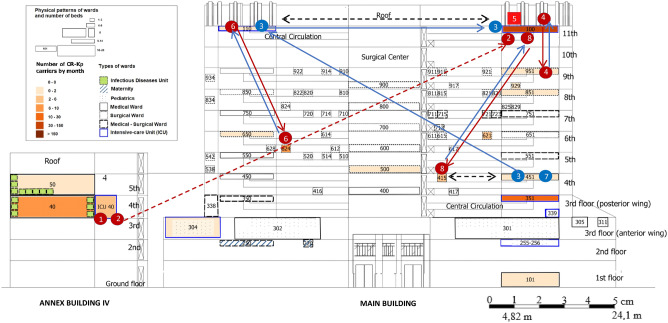
Space–time monthly distribution of patients harboring the carbapenem-resistant *K. pneumoniae* (CR-Kp) species complex and flow of cases (1–8) with polymyxin-carbapenem-resistant *K. pneumoniae* (PCR-Kp), by the hospital’s Geographic Information System^17^. Thematic hospital map in QGIS format (version 2.18, Open-Source Geospatial Foundation), federal tertiary hospital, Rio de Janeiro, December 2014 to August 2015^17^. The ward number is positioned in the center of its respective physical area. Patient numbers in red (ST437 PCR-Kp cases) or blue circles (ST11 PCR-Kp cases) ordered by the date of strain detection. *K. pneumoniae* complex isolate screened by the Vitek-2 system as PCR phenotype from case 5 (pink circle) was not preserved, but its AMR pattern (see Supplementary Table 2) was compatible with ST437 strains. The blue and red arrows represent the transfer of PCR-Kp infected cases before and after the detection of the PCR-Kp isolate, respectively. The dashed red arrow indicates that this patient was likely carrying PCR-Kp, although it had not yet been detected (see Table 1, PCR-Kp of cases 1 and 2 forms a subcluster of transmission). The dashed black arrow indicates that wards pertain to the same clinic or work as the same ICU. None of the cases had the opportunity for direct transmission to another case, considering the hospitalization unit and period. Superscript a: the number of patients in each ward or unit was counted monthly for the period of hospitalization after the first detection of CR-Kp complex.

Figure 3 shows a schematic diagram representing patients infected by PCR-Kp (7 cases: 1, 2, 3, 4, 6, 7 and 8), by unit and period of hospitalization, including case 5 information, in which the target isolate has not been preserved for further testing. The opportunities for transmission in ICU and non-ICU wards were investigated by the hospital's geographic information system (GIS) (Fig. 4), showing the spatial distribution of CR-Kp complex and the flow of cases infected by PCR-Kp.

### Complete report of index cases and characteristics of patients with target profiles

The complete report of the first two cases, who had close spatiotemporal links (index cases), and the summary of clinical and epidemiological characteristics of all patients infected by target PCR-Kp complex isolate are described in the Supplementary file (Complete Report of Index Cases and Supplementary Table 1). Three patients (cases 2, 3 and 6) had prior rectal colonization with *K. pneumoniae* complex displaying the target phenotypic profile and case 8 was previously colonized with carbapenem-resistant *Enterobacteriaceae* (CRE) (Supplementary Table 1). Urinary tract infection was responsible for half of the occurrences (n = 4), followed by ventilator-associated pneumonia (VAP, n = 2), catheter-related bloodstream infection (n = 1) and surgical site infection (n = 1)*.* A high proportion of the cases presented sepsis (6/8, 75%), progressing to an early (within four days of strain detection, in cases 1, 3, and 5) or hospital death (5/8, 63%).

### Antibiotic susceptibility phenotype, carbapenemase production, pulsed field gel electrophoresis (PFGE) and MLST genotypes of target PCR-Kp

Supplementary Table 2 shows the antimicrobial susceptibility profile of all preserved PCR-Kp isolates (n = 7). Unpreserved *K. pneumoniae* complex isolates (n = 5 strains) screened as PCR from cases 2, 4, 5 and 6 are also shown in this Table.

CCBH17440 and CCBH17428 were the only proven strains with an XDR pattern due to the susceptibility revealed to CZA only (single susceptible profile). The MIC values of CZA against these isolates were 0.5 mg/L. The MIC was highly elevated for most of the drugs tested, except for aminoglycosides (5/12, 42%) and tigecycline (9%, 1/11), to which few strains showed phenotypic susceptibility (Supplementary Table 2). All preserved strains had a positive carbapenemase inhibition test and amplified *bla*_KPC_, except CCBH 17724 (recovered from case 3), which did not amplify any carbapenemase gene investigated by conventional multiplex polymerase chain reaction (PCR), despite being positive for phenotypic detection of carbapenemase production in both hospital and research laboratories. These strains comprise three PFGE profiles and two MLST, ST437 (n = 5 strains from cases 1, 2, 4, 6 and 8) and ST11 (n = 2 strains, from cases 3 and 7) (Supplementary Fig. 1).

### Genomic features and phylogeny of clinical ST437 and ST11 PCR-Kp

**Figure 5:**
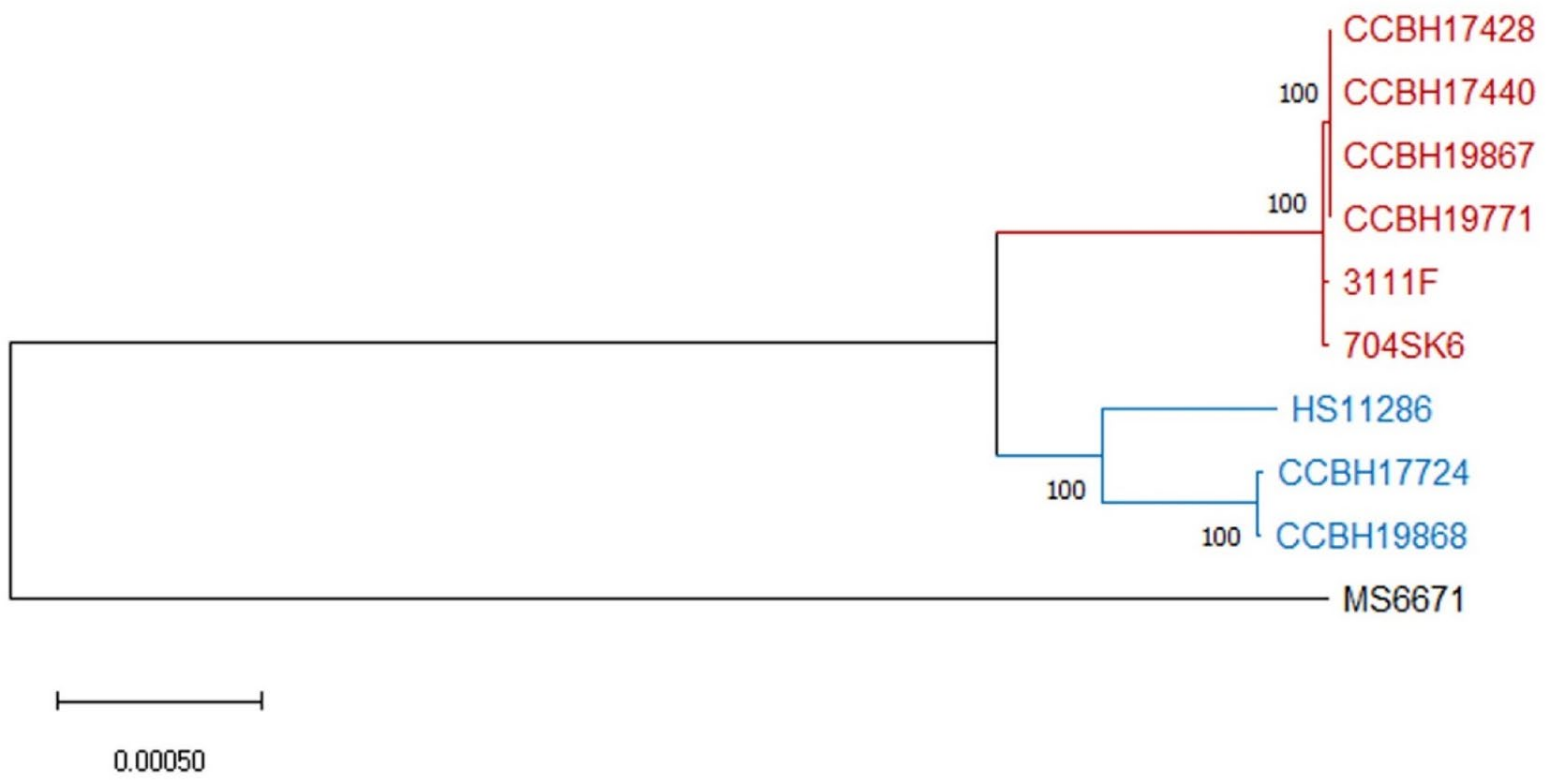
Phylogenetic inference. Neighbor-joining (NJ) distance tree representing phylogenetic relationships between polymyxin-carbapenem-resistant *K. pneumoniae* of the two distinct MLSTs, ST437 (red) and ST11 (blue), and publicly available genomic sequences 3111F, 704SK6, HS11286 and MS6671 (GenBank assembly accession numbers GCA_002251715.1, GCA_002211665.1, GCA_00240185.2 and GCA_001455995.1, respectively). MS6671 was selected as an outgroup. Numbers displayed in internal branches correspond to bootstrap values. The scale represents the NJ distance.

Supplementary Table 3 provides the genomic characteristics of each isolate. Strain ST437 CCBH19496 (case 4) was excluded from the analysis due to experimental problems. The phylogenetic tree and Mash distance show the close evolutionary relationships among strains from each ST and confirmed the clonal outbreak (Fig. 5 and Table 1). All strains have a strong match (Mash distance ≤ 0.02), and cases indexes’ ST437 isolates forming a subcluster (Mash distance < 0.0003). Very few genetic variations were found within ST437 isolates (Tables 1 and 2), but not within ST11 strains (Table 1), despite time differences between the first and last isolates in each clade.

**Table 1:**
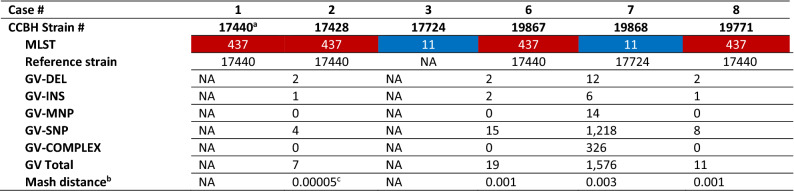
Genetic variations (GV) and Mash distance between polymyxin-carbapenem-resistant *K. pneumoniae* strains (ST437 in red and ST11 in blue).

Case number and respective strain in order of detection.

^a^Index strains; ^b^Ondov et al., 2016^39^; ^c^index strains form a subcluster of transmission (Mash distance < 0.0003)

*COMPLEX* combination of SNP and MNP, *DEL* deletion, *INS* insertion, *MLST* multilocus sequence typing, *MNP* multiple nucleotide polymorphism, *NA* not applicable, *SNP* single nucleotide polymorphism.

**Table 2:**
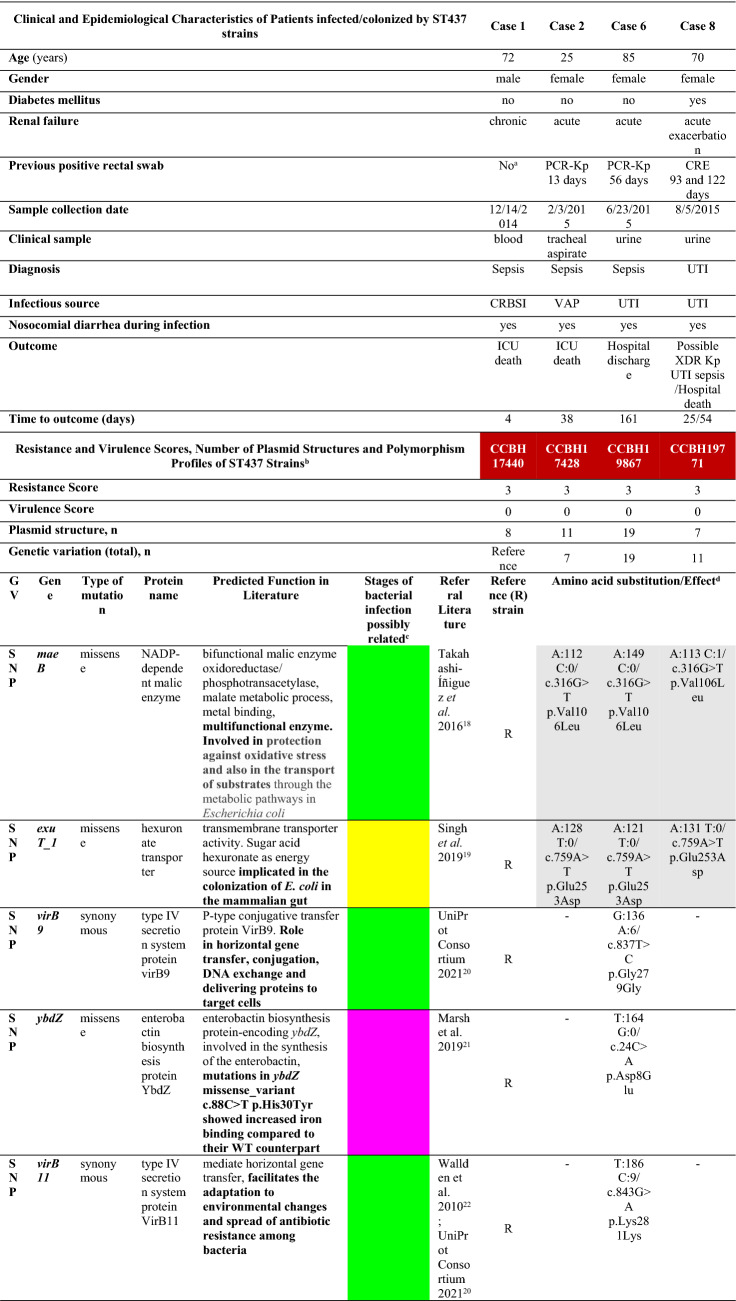

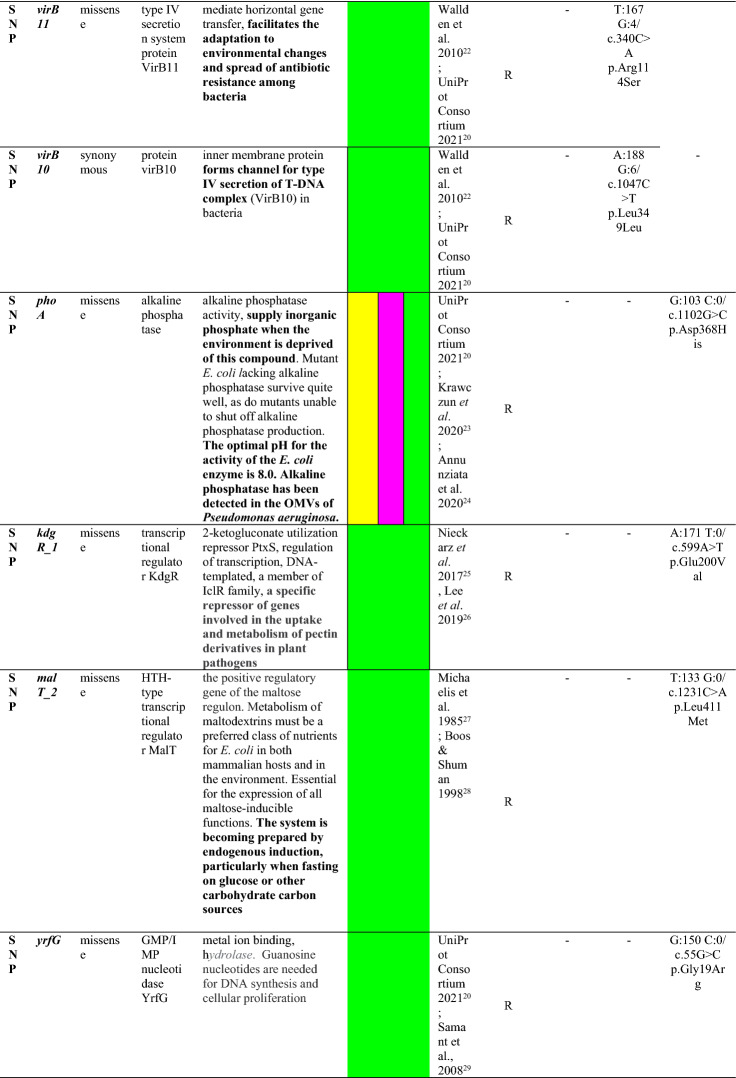
Clinical, epidemiological and genetic characteristics of ST437 polymyxin-resistant carbapenemase-producing *K. pneumoniae* strains and SNP variations possibly related to specific stages of bacterial infection.

Case number and respective strain in order of detection.

^a^Rectal swab negative for carbapenem-resistant Enterobacteriaceae (CRE) on 12/15/2014.

^b^Not discriminating mutations that identify hypothetical or undefined proteins.

^c^Specific stages of bacterial infection in which the mutated gene could be related, according to the referral literature, as: adherence and mucosal colonization (yellow), invasion and systemic infection (pink) and resistance, survival or proliferation (green).

^d^Same mutation is highlighted in gray color in Table cells.

*OMVs* outer membrane vesicles, *PCR-Kp* polymyxin-carbapenem-resistant *K. pneumoniae.*

Compared to the genome sequence of the same ST, retrieved from the National Biotechnology Information Center (NCBI, USA) (Fig. 5), our ST437 strains are closely related and have a strong match (Mash distances < 0.005) to ST437 *K. pneumoniae* 3111F, carrying the *mcr*-1 and *bla*_KPC-2_ genes, obtained from rectal swabs of a hospitalized patient in Porto Alegre city, southern Brazil, in July 2014^6^. In addition, *K. pneumoniae* 704SK6 encoding OXA-48 and CTX-M-15 from wastewater near Basel, Switzerland, in December 2015, has genetic profiles (Mash distances varying from 0.0046 to 0.0056) similar to those of our ST437 strains^30^. CCBH19868 and CCBH17724 have a strong match (Mash distance < 0.005) with a ST11 KPC-2-producing isolate (HS11286) collected from a sputum specimen of an inpatient in Shanghai, China, in 2011^31^, which is closely related to the worldwide-dominant CR-Kp clone ST258^31^.

### Hyperresistance and virulence profiles of clinical ST437 and ST11 PCR-Kp by whole-genome sequencing (WGS)

All organisms harbored several antimicrobial resistance (AMR) genes related to all antimicrobial classes (Supplementary Table 4) confirming the hyperresistant phenotype of these strains (Supplementary Table 2). The *bla*_KPC-2_ gene was present in all strains except ST11 CCBH17724 (case 3), classified as a carbapenemase producer due to positive carbapenemase-phenotypic test. However, all strains presented extended-spectrum beta-lactamase genes, which along with *ompK36GD* or *ompK35* porin mutations explain their high carbapenem resistance level. Polymyxin resistance was associated with *mgr*B truncation and the absence of *pmr*B in all isolates. See the complete AMR genetic profile and references in Supplementary Table 4 and Excel file 1.

We reported several virulence genes and features, including SNPs, according to the main biological characteristics predicted in the literature, possibly leading to specific stages of PCR-Kp infection (Supplementary Table 5, Excel file 1 and Table 2). The capsule (K) and O antigen loci of ST437 and ST11 isolates were predicted as KL36 or KL27 and O4 or O2 variant 2 (O2v2) with global identities of ≥ 99.88% and ≥ 98.43%, respectively, according to Kleborate(default settings). Index strains (CCBH17440 and CCBH17428) have a mucoid aspect, but the string-test performed only in these strains was negative, and no hypermucoviscosity genes were detected in the studied genomes. All strains present similar siderophores enterobactin and salmochelin (65% of sequence identity and 100% sequence coverage), and highly similar aerobactin receptor *iutA* (99–100% global identity), but no aerobactin gene was found. Complete yersiniabactin and incomplete genotoxin colibactin clusters (*clb*S was not detected*)* were found in ST11 CCBH19868 (case 7). The complete tellurite operon does not punctuate the virulence score but has been associated with hypervirulence, heavy metal resistance, infection, and resistance to stress induced by the indigenous gut microbiota during colonization. This operon was detected in all ST437 strains, but was found incomplete in ST11 members (Supplementary Table 5 and Excel file 1). ST11 CCBH19868 (case 7) has the highest virulence and resistance scores, while the other strains have zero virulence and maximum resistance scores.

### Plasmid structures of clinical PCR-Kp from CC258 ST437 and ST11

Plasmid types and incompatibility groups, with the exception of Col440I and Col(pHAD28), which were found in all samples, differentiated STs but were similar within the STs (Supplementary Tables 6 and 7). Therefore, all ST437 strains shared some plasmid contigs of different reference types and replicons: IncFIB(pNDM-Mar), IncHI1B(pNDM-MAR) and IncFIB(pKPHS1). The IncN_1 group was common in the majority of ST437 strains. The IncA/C2 plasmid was detected only in ST437 CCBH19867 (case 6), which had the highest number of plasmid contigs, types and replicons. Similarly, both ST11 strains shared some plasmid contigs of different types and replicons (Supplementary Table 7).

### Non-sustained antimicrobial combination synergy effect in the index strains

Meropenem combined with colistin decreased the bacterial burden by ≥ 2 log_10_ cfu/mL compared to the most active single agent at 24 h against both index strains tested samples. The combination failed to meet the definition of synergy due to achieving < 1 log_10_ cfu/mL reduction from the initial inoculum at 24 h. The addition of daptomycin did not seem to improve the bactericidal activity of meropenem plus colistin against either of the isolates (Supplementary Fig. 2). Other antimicrobial combination therapies were not tested.

### Untreatable infections

Both index cases (cases 1 and 2) fulfilled the criteria for untreatable infection caused by ST437 strains due to the unavailability of active drugs to treat their systemic infections. Similarly, case 5 was diagnosed with an untreatable infection caused by an unpreserved *K. pneumoniae* complex screened as PCR recovered from bronchoalveolar lavage (> 10^6^ CFU), displaying non-susceptibility to all antibiotics among all categories recommended to treat VAP.

## Discussion

In this full report, we describe the epidemic profile of PCR-Kp in which two index ST437 strains characterized as a PDR profile proved later to be susceptible to one of the novel cephalosporin/beta-lactamase inhibitor combinations that was not licensed at the time of study. Although uncommonly reported^32^, there were no drugs approved to treat some of these infections globally at the time of these occurrences.

These strains caused severe systemic infections, with the index ST437 strains showing non-sustained in vitro synergistic effects of the combination therapy most commonly used for CR-Kp^8^. These factors, together with the epidemiological context and significant genetic factors found in these representatives of CC258, contributed to the warning about this successful pathogen with highly resistant profiles and basic virulence, triggering rapid and difficult-to-treat infections, mainly fatal or incurable in a Brazilian sentinel hospital^8^.

The availability of sequenced genomes was fundamental for understanding the spread of clinical PCR-Kp in the surveyed hospital and to conclude this report^8^. During nine-months, in this endemic state of highly elevated MICs of meropenem among CR-Kp, it was possible to detect the clonal aspect and confirm the outbreak by a higher resistance profile (PCR-Kp), with a significant proportion of cases reaching the definition of intractable infections (38%, 3/8), early death (within four days after strain detection, 38%, 3/8) and hospital death (63%, 5/8).

In addition to the overuse of antimicrobials to treat nosocomial infections, which are the principal drivers in the development of drug-resistant pathogens^33^, as exemplified by the complete report of the index cases (Supplementary file), the temporal and spatial occurrences between cases and the clonal relatedness between strains corroborate the cross-transmission of extremely resistant *K. pneumoniae*. All patients infected with clonal ST437 PCR-Kp subsequently used the same bed in the infectious disease (ID)-ICU (subcluster of transmission) or the nearby bed at the adult MS-ICU during the same period or with an interval of days, a month or two. Patients infected by ST11 strains were admitted to the same clinical ward five months apart. Strains of both STs circulated concomitantly in the MS-ICU, surgical and general medicine wards during this study period, or could be the hospital reentrance of closely related ST11 strains^34^. In fact, ST437 and ST11 CR-Kp were previously described as prevalent in hospitals in Rio de Janeiro, Brazil, with low level (MIC50/90: 2/4 μg/mL) colistin co-resistance in 14%^35^, contrasting with higher MICs by 40% in our study.

Some of the cases had several opportunities for transmission due to prolonged hospital stays. However, none of the cases transmitted PCR-Kp directly to each other, which was demonstrated through the hospital spatial methodology^17^. Therefore, silent colonization is likely during this outbreak^36^, but these may also indicate infection control. However, the complex dynamics of *K. pneumoniae* transmission cannot be investigated without massive rectal swab surveillance and preservation of rectal swab isolates^36,37^. In addition, the Vitek-2 system tends to underestimate MICs for polymyxin resistant isolates and is no longer recommended in clinical settings^38^. Despite these limitations, although more occurrences would be expected, genetic tracking of clinical samples was enough to document the outbreak and patient-to-patient transmission, by confirming the epidemiological and genetic link between isolates.

ST437 genomes displayed reciprocal SNP occurrences below the threshold of 16 SNP^39^ for interhospital transmission^40,41^. Therefore, we confirmed the same transmission cluster among patients infected by ST437 PCR-Kp, which extended their occurrences throughout the entire period of surveillance. The first two ST437 isolates even formed a subcluster with a Mash distance far below the cut-off of 0.0003^42^, corroborating the initial epidemiological hypothesis of a common source while these patients occupied the same ID-ICU bed. The substantial similarity between the ST11 strains (Mash distance = 0.003) indicates a common ancestor for these bacteria. However, the higher genetic polymorphism among ST11 strains at five months apart, compared to the small genetic variability among ST437 strains over eight months, suggests a different source of ST11 PCR-Kp acquisition.

The comparison of our ST437 strains with the genomes from the same ST retrieved from NCBI, one recovered from a rectal swab sample of a patient in southern Brazil in 2014^6^ and the other from a wastewater sampled in Switzerland in 2015^30^, contributes to discussing the origin or adaptation of ST437 strains in the gastrointestinal tract, but possibly from our hospital environment^43^. Environmental contamination is likely since some of the reported cases had unwieldy diarrhea while colonized and infected with ST437 and ST11 PCR-Kp. Therefore, the lack of sampling environmental surface and healthcare workers’ hands are significant limitations of our study^36^.

Regarding diarrhea, we did not find enterotoxigenic genes encoded in the genome sequence of our samples, as previously detected in *K. pneumoniae* and other members of the *Enterobacteriaceae* family by primer-specific PCR methods^44^. *K. pneumoniae* colonization has been implicated in chronic diseases of the gastrointestinal tract, including inflammatory bowel disease and colorectal cancer^45^. Moreover, in animal models, the transmission of *K. pneumoniae* requires contact with feces, and the supershedder phenotype, with increased efficient transmission, occurs and persists while on antibiotic treatment^37^.

Types of infection correspond to high rates of gastrointestinal colonization and the prevalence of hospital-acquired infections caused by CR-Kp^46^. Although the number of cases was too low, we observed the early death over late or no death in patients without previous rectal colonization (67% versus 0%). This observation should be further investigated, as well as its relationship with the source of infection, since a more severe infection would be expected in patients who have direct contact with an infectious agent of exogenous origin, rather than endogenous origin, such as the gastrointestinal tract.

ICU admission, tracheal cannula and prior exposure to carbapenem antibiotics have been described as risk factors for infection with XDR CR-Kp susceptible to polymyxin^47^. In turn, previous treatment with colistin, preceding colonization of resistant *K. pneumoniae*, and a Charlson score of ≥ 3 were correlated with colistin-resistant KPC-producing *K. pneumoniae* infection^48^. All these factors were invariably or variably present in our reported cases, typifying the burden of AMR, affecting primarily immunocompromised patients, but also an young woman who became ill and required hospitalization.

The significant variability of AMR phenotypes found in *K. pneumoniae* complex isolates may indicate great diversity in MLST types throughout the institution. In fact, among 30 unselected clinical and surveillance *K. pneumoniae* isolates from inpatients ˗ 18 CR-Kp isolates preserved during 2015–2016—we found 23 MLST (eight new STs), and 19 PFGE types among 20 tested (data not shown). This high genetic variability may indicate high-level horizontal genetic transfer^49^ in a pressured hospital environment due to high antimicrobial consumption.

Resistance genes detected against different antimicrobial classes corroborate the resistance profile of the strains. However, predicting the phenotype of antimicrobial susceptibility from bacterial genetic data is challenging, because it is based on the quality and completeness of the existing information about the genomic determinants of resistance^50,51^. Despite the enormous advances in bioinformatics^20,50,52,53^, it was noted that no database includes complete phenotypic profile data associated with the AMR gene sequence^50,51^. The resistance phenotype conferred by the presence of some genes must be inferred from exhaustive searches in the literature (Supplementary Table 4 and Supplementary Table 4A)^50,51^.

The performance of WGS to predict beta-lactam, fluoroquinolone and aminoglycoside susceptibility has been considered excellent for *K. pneumoniae*^50^. Other carbapenemases have been described in *K. pneumonia* as well^54,55^, but we were not able to confirm any carbapenemase encoded in an ST11 strain by manual curation, despite the evidence of positive carbapenemase screening tests, which may indicate a novel carbapenemase. ST11 strains have *aac(6')Ib-cr* and *aadA2* genes but are reported to be inversely susceptible to gentamycin but nonsusceptible to amikacin^50^. We did not detect the plasmidial *mcr-*1 gene in the analyzed genomes, but the genes *mgr*B and the component system *pmr*A*/pmr*B were truncated or absent in all strains, which are related to the genetic mechanisms associated with polymyxin B/E resistance^56,57^. Val*130* to Ala mutation in *oqx*R has been reported in both tigecycline-nonsusceptible and tigecycline-susceptible strains^58^, but the lack of knowledge about the expression levels of the efflux pump genes detected may have precluded the identification of this resistance mechanism^59^.

Even more challenging is choose to correlate virulence gene functions inferred from the literature as one of our approaches (Supplementary Table 5 and Supplementary Table 5A), because precise predictions of gene functions may not be possible due to the complexities in the subjacent genetic mechanisms not yet completely comprehended^52^. Despite the limitations, our purpose was only to raise hypotheses, through the descriptive comparison of bacterial genomics with the clinical and epidemiological characteristics of affected patients. Despite the severity of infections, most of the genetic structures found in PCR-Kp are related to resistance, survival, and proliferation in the revised literature (Supplementary Table 5 green color). The same pattern of genetic functions seems to have predominated in SNP variations among ST437 strains (Table 2, green color), although most mutations are missense, and the resulting protein structures and functions were not investigated in this study^60^.

Virulence genes and other features found in PCR-Kp indicate several putative basic skills to invade tissue and persist in the hospital environment. These abilities were related in different strains to the presence of genetic determinants of the capsule, adhesins, surface attachment, biofilm formation, efficient bacterial gastrointestinal colonization, siderophores, outer membrane vesicles, signaling, secretion, transport, efflux systems, regulation, endotoxin, serum resistance, immune evasion, intracellular survival, heavy metal resistance and AMR (references in the Supplementary files), imposing additional challenges for the treatment and control of nosocomial infections caused by PCR-Kp. Most of these factors are common to all *K*. *pneumoniae* and conserved in the chromosome as core genes^61,62^.

Among our strains, of particular importance is the additional encoding siderophore system, namely yersiniabactin (Ybt)^62,63^, which enhances the ability to scavenge iron from its surrounding environment for rapid growth and subsequent invasion, and genotoxin colibactin clusters^45^, detected in CCBH 19868 only. These genes are encoded by loci usually located within a mobilized genetic element detected in this strain (ICE*Kp*10), which is a concern due to its potential of being mobilized independently between enterobacteria by horizontal gene transfer or being stable within *K. pneumoniae* lineages by vertical inheritance^63–65^.

Last but not least, we would like to emphasize the importance of having in mind not only the presence or absence of a given gene, but also if it encodes a full-length protein and what clinical implication it may have^52^. Published resistance and virulence scores are not intended to predict clinical virulence or antibiotic resistance^52^. However, our findings related to the ST11 CCBH19868 strain are at least intriguing. It was ranked with a comparatively higher virulence score, but detected with an incomplete colibactin gene cluster^45,66^, causing UTI only in a 65-year-old man with diabetes mellitus and chronic renal failure, who was not admitted to the ICU, discharged early and treated on an outpatient basis. Consequently, more studies are needed to compare the clinical and epidemiological findings of infected patients with bacterial genetic markers of virulence, resistance, and pathogenicity.

These lineages have a selective advantage in hospitals, where antimicrobial consumption is high and the environment has abundant opportunities for cross-transmission of microorganisms, along with the potential for dissemination of resistance and virulence genes through transmissible plasmids. The ability of resistance and virulence plasmids to be maintained in *K. pneumoniae* lineages suggests that once established in clones associated with hospital outbreaks, they may become relatively stable^61^. The similarities and differences in resistance, virulence, plasmid profiles and genetic polymorphism between our strains of the same clade over nine months (Supplementary Tables 4–7 and Table 1) agree with this observation. Two distinct missense mutations in the *mae*B gene (c.316G > T p.Val106Leu), encoding an NADP-dependent malic enzyme, and *exu*T_1 **(**c.759A > T p.Glu253Asp), coding for a hexuronate transporter, related to resistance, survival or proliferation (green color in Table 2) and adherence and mucosal colonization (yellow color in Table 2)^18,19^ are shared among all ST437 strains (CCBH17428, CCBH19867, and CCBH19771) compared to the reference ST437 strain (CCBH17440), suggesting that these mutations are not random. Since the study period, these descendant lineages likely emerged as a persistent hyperresistant and virulent form of *K. pneumonia* in the study setting^17^.

Increased resistance and relatively low virulence are probably the compensatory mechanisms required due to the burden associated with the extensive use of antibiotics in which bacteria act to increase fitness and resistance to the surrounding environment. Considering that hospitalized patients are generally immunocompromised with underlying conditions and invasive procedures, bacteria do not need to raise virulence rather than resistance to overcome antimicrobial damage with which these patients are usually treated. In many circumstances, bacteria are transported accidentally and directly into the bloodstream or the infectious focus by an invasive procedure and do not need to break down barriers to invasion, but only survive in the new environment. Under these circumstances, even previous immunocompetent patients are in danger. Therefore, in addition to the patient’s comorbidities, the source and route of infection and the microbial load are essential points to be considered in studying the genetic structure of bacteria and its association with deadly hospital infection. Moreover, many host, environmental, and bacterial factors affecting the virulence phenotype of *K. pneumonia* remain to be identified^67^. Experimentation in animals is necessary for characterizing the invading pathogen and the host response^37^; this type of study has begun to yield information about *K. pneumoniae* biology and its interaction with the host^37^.

The definition of untreatable infections was arbitrary based on clinical and laboratory parameters for surveillance purposes, setting up another limitation. In clinical practice, several interrelated factors of patients, the quality of medical care and the pharmacological properties of drugs not considered in this study may interfere with untreatable infection. Time-kill analysis typically provides descriptive information on pharmacodynamics and complicates the translation of in vitro results to the killing performance of antimicrobial agents^68,69^. However, the literature corroborated our findings that infection with PCR-Kp has not benefited from this combination^70^. Therefore, regimens containing drugs with novel mechanisms of action are necessary for treatment. The investigation of the triple combination of colistin, meropenem and daptomycin, a lipopeptide agent that carries no Gram-negative activity, was advocated by in vitro data showing that it works synergistically against resistant *A. baumannii*^71^.

Among new drugs, we could assess only CZA against CCBH17440 and CCBH17428. However, it is possible that drugs such as meropenem-vaboractam, imipenem-cilastatim-relebactam, plazomicin, eravacycline, omadacycline, aztreonam-avibactam or cefiderocol might have an effect, or other noninvestigated combination therapies^72,73^. Susceptibility to CZA was only tested in vitro, and the emergence of resistance to CZA during monotherapy mitigated the initial promising results^72^. Clinical experiences of CZA combined with colistin or amikacin to treat infections caused by XDR *Enterobacteriaceae* have brought greater attention, presenting a clinical success rate^74,75^. However, dialysis patients, accounting for 86% of our patients, were at risk of a worse prognosis^75^.

In conclusion, this report shows what typically happens in hospitals and may help rethink infection control strategies, while advising on access to new antimicrobials for the treatment of PCR-Kp infection. Daily monitoring of all microbiological results to detect early emerging resistant phenotypes, guiding infection surveillance and control, is an important strategy, but we cannot determine how this contributed to containing the intrahospital spread of PCR-Kp during the study. The infection control implemented was insufficient, as described in other outbreaks caused by PCR-Kp^76^, and new cases of colonization and infection have continued to be reported^17^. The lack of drugs to treat PCR-Kp infections likely increases the risk of bacterial spread^33,37^. Controlling cross-transmission and nosocomial infection by well-equipped, developed, virulent and extensively drug-resistant bacteria likely requires strict antimicrobial stewardship and infection control measures beyond the standard^36^. Hospital-acquired diarrhea in five of our PCR-Kp cases may indicate its containment as part of nosocomial infection control measures for highly resistant and virulent bacteria that usually colonize the gastrointestinal tract.

Taking everything above into consideration, in addition to the importance given in the literature to the confluence of known hypervirulence features in highly resistant bacteria, any *K. pneumoniae* with a resistance score of three should be taken seriously in hospitals. The general abilities to resist the bactericidal activity of the serum, and thus survive in the bloodstream, and proliferate under antibiotic pressure by themselves represent sufficient traces of virulence. Although most of our patients are immunocompromised, slight differences in bacterial genome, source and types of infection, and even in prognosis are attractive for future clinical and microbiological research in hospitals.

## Materials and methods

### Hospital-wide surveillance of *K. pneumonia*e species complex with concomitant resistance to carbapenem and polymyxin

The surveillance was initiated in a 450-bed federal tertiary hospital, located in Rio de Janeiro, after the first detection of *K. pneumonia*e complex strains with a PDR phenotype (CCBH17440 and CCBH17428) in index cases, who occupied the same bed in the ID-ICU with a five-day interval. During the investigation period, from December 2014 to August 2015, we prospectively monitored the antimicrobial susceptibility profiles of all *K. pneumoniae* species complex recovered in clinical and surveillance samples of hospitalized patients. Clinical samples were collected from the routine service of attending physicians guided by the microbiological protocol implemented throughout the institution by the Hospital Infection Control Committee (HICC)^17^. Active surveillance with rectal swabs was performed weekly or every two weeks on a routine basis in all ICU patients, and high-risk patients admitted to nonintensive care wards as described previously^17^. We followed ORION statements in this study report^77^ and all methods were performed in accordance with the relevant guidelines and regulations.

We classified the susceptibility profile of all *K. pneumoniae* complex isolates into non-multidrug-resistant (non-MDR), multidrug-resistant (MDR), and possible XDR or possible PDR profiles, according to the criteria described in Magiorakos et al. 2012^16^. Clinical isolates of *K. pneumoniae* complex with an initial PDR or XDR profile and nonsusceptibility to carbapenems and screened positive for polymyxin resistance (target isolates) were preserved for additional microbiological tests. CCBH17440 and CCBH17428 were the only strains tested against ceftazidime-avibactam (CZA), an advanced generation cephalosporin. Target isolates from rectal swabs could not be preserved during the study period due to the additional workforce required in the hospital microbiology laboratory.

To determine the monthly incidence density of clinical *K. pneumoniae* complex phenotypes per 1000 patient-days, we considered only newly detected isolates with the specific phenotype (non-MDR, MDR, possible XDR or possible PDR) per month, excluding *K. pneumoniae* complex isolates from the same biological sample collected on the same day and all rectal swab isolates.

The space-temporal distribution was also investigated based on patients with a specific phenotype (CRKp complex) counted monthly from the day of the first detection to the date of hospital discharge or death, using the same method described previously, in which the hospital GIS demonstrated the flow of patients with PCR-Kp^17^. Institutional review boards approved this study with a waiver of informed consent. Although the researchers did not interfere with the clinical investigation or hospital surveillance program, all investigations and results were reported to the HICC in a timely manner. The hospital infection control program was actively maintained and reinforced throughout the study period, following national and international guidelines^17,78,79^.

### Bacterial identification and susceptibility testing

The bacterial identification and antimicrobial susceptibility tests performed in the hospital microbiology laboratory were carried out using the Vitek-2 system (BioMérieux, France), including those recovered from rectal swabs, which were directly inoculated onto selective chromogenic media (CHROMagar Co., Paris, France) supplemented with meropenem for the detection of CRE. Rectal swabs were also plated on MacConkey agar (Oxoid, Lawrence, USA) to detect ESBL-producing *Enterobacteriaceae*, especially from pediatric units. Screening for carbapenemase production was performed with phenylboronic acid, ethylenediaminetetraacetic acid and cloxacillin as recommended^80,81^ and previously described^17^. All preserved clinical *K. pneumoniae* complex strains with the target antimicrobial susceptibility profile (n = 7, CCBH17440, CCBH17428, CCBH17724, CCBH19496, CCBH19867, CCBH19868, and CCBH19771) had the species confirmed by classical biochemical tests in Laboratório de Pesquisa em Infecção Hospitalar, Oswaldo Cruz Institute, FIOCRUZ. Antibiotic susceptibility testing (Supplementary Table 2) was also confirmed using broth microdilution, Etest (Biomérieux) and disk diffusion (Oxoid; Hampshire, UK) methods according to the Clinical Laboratory Standards Institute (2016) and European Committee on Antimicrobial Susceptibility Testing (2016) criteria^82,83^. More information on the methods used in antimicrobial susceptibility tests is described in the footnotes of Supplementary Table 2**.**

### Detection of carbapenemase genes and molecular typing of target clinical PCR-Kp

We performed an in-house multiplex PCR assay to detect commonly described carbapenemase genes, *bla*_KPC, _*bla*_NDM_, and *bla*_OXA-48-like_, in *K. pneumoniae*. To assess the genetic relatedness of the isolates, we carried out PFGE of XbaI digestion genomic DNA^84^ and MLST according to a protocol previously described^85^.

### Whole-genome sequencing, genomic analysis, and phylogeny of target clinical PCR-Kp

The complete genomes were extracted using a QIAamp DNA Blood Mini Kit (Qiagen, Germany) and sequenced using an Illumina MiSeq platform (Illumina Inc., USA). The genomic library was constructed by transposon tagmentation with the Nextera XT DNA Sample Prep kit (Illumina Inc). Sequence reads were then trimmed and filtered using a Phred score > 20. The software A5-miseq, an updated pipeline to assemble microbial genomes from Illumina MiSeq data, was used for de novo assembly^86^.

The assembled scaffolds (CCBH17440, CCBH17428, CCBH17724, CCBH19496, CCBH19867, CCBH19868, and CCBH19771) and publicly available genomic sequences (HS11286, MS6671, 704SK6, 3111F) were automatically annotated with rapid prokaryote genome annotation (PROKKA) < https://github.com/tseemann/prokka >^87^ as follows: *prokka kingdom Bacteria genus Klebsiella—species pneumoniae*. Annotated assemblies in GFF3 format-containing the assembled sequences (produced by Prokka) was used to predict shared orthologous protein-coding genes between all bacterial samples, and obtain a multiple sequence alignment of concatenated core genes (4,049 genes encoded in at least 99% of the analyzed genomes), with the rapid large-scale prokaryote pan-genome analysis (Roary) pipeline < https://github.com/sanger-pathogens/Roary >^88^, employing MAFFT (https://doi.org/10.1093/nar/gkf436, https://doi.org/10.1093/molbev/mst010) to align the sequences.

In silico MLST was carried out using specific platforms (https://cge.cbs.dtu.dk/services/MLST)^89^. Phylogenetic tree reconstruction based on core genome of the analyzed samples was obtained with Molecular Evolutionary Genetics Analysis (MEGA) software version X < https://www.megasoftware.net >^90^, applying the neighbor-joining algorithm^91^. Evolutionary distances were computed using the maximum composite likelihood method^92^, expressed as the number of base substitutions per site, and 500 bootstrap replicates were applied for statistical evaluation. The distance between genomic sequences was estimated with Mash^42^, and single nucleotide polymorphisms (SNPs) were analyzed with Snippy (Seemann T, Snippy, Github https://github.com/tseemann/snippy), applying default parameters.

Genomic and plasmid-mediated AMR and virulence genes in samples CCBH17440, CCBH17428, CCBH17724, CCBH19867, CCBH19868 and CCBH19771 were detected with ABRicate (Seemann T, *Abricate*, Github https://github.com/tseemann/abricate) with default parameters, employing the following databases and software: NCBI AMRFinderPlus (https://www.ncbi.nlm.nih.gov/pathogens/antimicrobial-resistance/AMRFinder)^93^, Comprehensive Antibiotic Resistance Database (CARD) (http://arpcard.mcmaster.ca)^94^, Resfinder^95^, Antibiotic Resistance Gene-ANNOTation (ARG-ANNOT)^96^, Virulence Factor Database (VFDB)^97^, PlasmidFinder^98^, EcOH database^99^, and MEGARes 2.00 MEGARes (meglab.org)^100^. Additionally, samples were screened for resistance/virulence genes using the Institute Pasteur MLST database.

(https://bigsdb.pasteur.fr/klebsiella/klebsiella.html) and Kleborate^52^. Putative plasmids inferred by PlasmidFinder^98^ were confirmed with Platon^101^, by inspecting draft assemblies and characterizing contigs.

We investigated the presence of *pmr*A/B, *pho*P/Q, *mgr*B and *mcr*-1 genes related to polymyxin resistance^102^. To confirm the absence of the *pho*P/*pho*Q regulator *mgr*B-gene, predicted by PROKKA, we scanned each scaffold, searching for genomic regions similar to *K. pneumoniae* strain 342's *mgr*B-coding protein (SwissProt registry number B5XQ45) with BLAST version 2.9.0 +  < https://ftp.ncbi.nlm.nih.gov/blast/executables/blast + >^103^, with the following command-lines and parameters: *makeblastdb -in 'scaffolds_fasta_file' -dbtype nucl -out 'database_name'*; *tblastn -outfmt 4 -query'mgrb-gene_fasta_file' -db 'database_name' -out*^104^*'output_file_name'*. Alignment results were visually inspected. Resistance and virulence scores were reported according to Lam et al., 2021^52^. We also descriptively correlated resistance and virulence genes, including those related to genetic variation (SNP), with their respective protein names, predicted functions or main biological characteristics possibly related to stages of bacterial infection, according to the UniProtKB database^20^ and reference literature (references in Excel File 1), to improve the understanding of PCR-Kp strain infection.

### Antimicrobial synergy testing of index PCR-Kp strains

Time–kill studies performed in the first two isolates with a profile initially classified as PDR (CCBH17440 and CCBH17428) were performed using a 24-well microwell plate containing cation-adjusted Muller Hinton Broth (CAMHB, Difco, Detroit, MI) as growth media. Each plate was inoculated with either isolate to target initial inoculums of ~ 1 × 10^6^ cfu/mL, and a combination of colistin at 16 mg/L (0.5× MIC of both organisms) and meropenem at 49 mg/L (*f*Cmax of meropenem 1 g) was evaluated against each strain. Daptomycin at 9.39 mg/L (*f*Cmax of daptomycin 6 mg/kg) was added to investigate the potential additional benefit compared to meropenem plus colistin alone. Broth samples were taken at 0, 4, 8 and 24 h, serially diluted in sterile normal saline, and plated on tryptic soy agar (TSA) (Difco, Detroit, MI) using spiral platter. The plates were incubated for 24 h at 35 °C for colony enumeration. Time-kill curves were generated by plotting bacterial CFU/mL against each time point. Synergy was defined as a > 2 log_10_ cfu/mL reduction compared to the most active single agent of the combination while also achieving ≥ 1 log_10_ cfu/mL reduction from the initial inoculum at 24 h. The method is in accordance with CLSI, 2020^105^ and is the same method used in previously published experiments^106,107^. The quality control strains used were *Escherichia coli* ATCC^®^ 25922 and *K. pneumoniae* ATCC^®^ 700603^105^.

### Untreatable PCR-Kp infections

Moreover, we performed a chart review of all hospitalized patients harboring *K. pneumoniae* with the investigational antimicrobial susceptibility pattern. Untreatable infection was arbitrarily defined for surveillance purposes as any systemic monomicrobial infection caused by possible PDR or XDR *K. pneumoniae* with the following features: susceptible drugs are not recommended for the site of infection or not available in the country market and/or infections possibly forming biofilms, that cannot be removed surgically or by device withdrawal, and/or antagonism or non-synergistic action was evidenced by any combination therapy synergy testing.

### Conference presentation

This study was partly presented as a poster abstract at IDWEEK 2016, which was published in https://doi.org/10.1093/ofid/ofw172.1558.

### Ethics approval and consent to participate

This study was approved by the FIOCRUZ and HFSE Ethics Committees (CAAE: 60493516.6.0000.5248 and CAAE 60493516.6.3001.5252, respectively) with a waiver of informed consent.

## Supplementary Information

Supplementary Tables.

Supplementary Information.

## Data Availability

The datasets used and/or analyzed during the current study are available from the corresponding author on reasonable request. BioProject accessions PRJNA336378 (CCBH17440) and PRJNA678746 (other strains). GenBank Assembly Accession: GCA_001715215.1, GCA_017565915.1, GCA_017565865.1, GCA_017565945.1, GCA_017566015.1, GCA_017565885.1.

## Abbreviations

AMR: Antimicrobial resistance
*bla*: Beta-lactamase
CC258: Clonal complex 258
CCBH: Culture collection of hospital bacteria
CFU: Colony forming unit
CRE: Carbapenem-resistant *Enterobacteriaceae*
CR-Kp: Carbapenem-resistant *K. pneumoniae*
CTX-M: Cefotaximase-Munich
CZA: Ceftazidime-avibactam
DNA: Deoxyribonucleic acid
GIS: Geographic Information System
HICC: Hospital Infection Control Committee
hvKp: Hypervirulence *K. pneumoniae*
ICE: Integrative conjugal elements
ICEKp10: Integrative conjugative element 10
ICU: Intensive care unit
ID-ICU: Infectious disease ICU
KPC-2: *K. pneumoniae* Carbapenemase 2
MDR: Multidrug resistant
MIC: Minimum inhibitory concentration
MLST: Multi-locus sequences type
MS-ICU: Medical-surgical intensive-care unit
NCBI: National Biotechnology Information Center
NDM-1: New Delhi metallo-beta-lactamase 1
*ompK*: Outer membrane protein K
ORION: Outbreak Reports and Intervention Studies of Nosocomial infection
OXA-48: Oxacillinase-48-like carbapenemases
PCR: Polymerase chain reaction
PCR-Kp: Polymyxin-carbapenem-resistant *K. pneumoniae*
PDR: Pan-drug resistant
PFGE: Pulsed field gel electrophoresis
SNP: Single nucleotide polymorphism
ST: Sequences type
USA: United State of America
VAP: Ventilator-associated pneumonia
WGS: Whole-genome sequencing
Ybt: Yersiniabactin
XDR: Extensively-drug resistant

## References

[1] Cox JA (2017). Antibiotic stewardship in low- and middle-income countries: The same but different?. Clin. Microbiol. Infect..

[2] Paczosa MK, Mecsas J (2016). *Klebsiella pneumoniae*: Going on the offense with a strong defense. Microbiol. Mol. Biol. Rev..

[3] Queenan AM, Bush K (2007). Carbapenemases: The versatile beta-lactamases. Clin. Microbiol. Rev..

[4] Bartolleti F (2016). Polymyxin B resistance in carbapenem-resistant *Klebsiella pneumoniae*, Sao Paulo, Brazil. Emerg. Infect. Dis..

[5] Aires CAM (2017). Emergence of the plasmid-mediated mcr-1 gene in clinical KPC-2-producing *Klebsiella pneumoniae* sequence type 392 in Brazil. Antimicrob. Agents Chemother..

[6] Dalmolin TV, Martins AF, Zavascki AP, de Lima-Morales D, Barth AL (2018). Acquisition of the mcr-1 gene by a high-risk clone of KPC-2-producing *Klebsiella pneumoniae* ST437/CC258, Brazil. Diagn. Microbiol. Infect. Dis..

[7] Marchaim D (2011). Outbreak of colistin-resistant, carbapenem-resistant *Klebsiella pneumoniae* in metropolitan Detroit, Michigan. Antimicrob. Agents Chemother..

[8] Gomes MZR (2016). Clonal pan or extensively drug-resistant KPC-2-producing ST437 *Klebsiella pneumoniae* causing untreatable infections evidenced by in vitro synergy testing. Open Forum Infect. Dis..

[9] Avgoulea K (2018). Characterization of extensively drug-resistant or pandrug-resistant sequence type 147 and 101 OXA-48-producing *Klebsiella pneumoniae* causing bloodstream infections in patients in an intensive care unit. Antimicrob. Agents Chemother..

[10] da Silva KE, Thi Nguyen TN, Boinett CJ, Baker S, Simionatto S (2020). Molecular and epidemiological surveillance of polymyxin-resistant *Klebsiella pneumoniae* strains isolated from Brazil with multiple mgrB gene mutations. Int. J. Med. Microbiol..

[11] Heiden SE (2020). A *Klebsiella pneumoniae* ST307 outbreak clone from Germany demonstrates features of extensive drug resistance, hypermucoviscosity, and enhanced iron acquisition. Genome Med..

[12] Banerjee T, Wangkheimayum J, Sharma S, Kumar A, Bhattacharjee A (2021). extensively drug-resistant hypervirulent *Klebsiella pneumoniae* from a series of neonatal sepsis in a tertiary care hospital, India. Front. Med. (Lausanne).

[13] Jin X (2021). Resistance evolution of hypervirulent carbapenem-resistant *Klebsiella pneumoniae* ST11 during treatment with tigecycline and polymyxin. Emerg. Microbes Infect..

[14] Lai YC, Lu MC, Hsueh PR (2019). Hypervirulence and carbapenem resistance: two distinct evolutionary directions that led high-risk *Klebsiella pneumoniae* clones to epidemic success. Expert Rev. Mol. Diagn..

[15] Xie M (2021). Clinical evolution of ST11 carbapenem resistant and hypervirulent *Klebsiella pneumoniae*. Commun. Biol..

[16] Magiorakos AP (2012). Multidrug-resistant, extensively drug-resistant and pandrug-resistant bacteria: An international expert proposal for interim standard definitions for acquired resistance. Clin. Microbiol. Infect..

[17] da Silva PP (2021). Geographical information system and spatial-temporal statistics for monitoring infectious agents in hospital: A model using *Klebsiella pneumoniae* complex. Antimicrob. Resist. Infect. Control.

[18] Takahashi-Íñiguez T, Aburto-Rodríguez N, Vilchis-González AL, Flores ME (2016). Function, kinetic properties, crystallization, and regulation of microbial malate dehydrogenase. J. Zhejiang Univ. Sci. B.

[19] Singh B (2019). Molecular and functional insights into the regulation of d-galactonate metabolism by the transcriptional regulator DgoR in *Escherichia coli*. J. Bacteriol..

[20] Consortium, T. U. UniProt: The universal protein knowledgebase in 2021. *Nucleic Acids Res.* **49** (2021). 10.1093/nar/gkaa1100 PMC7778908 33237286

[21] Marsh JW (2019). Evolution of outbreak-causing carbapenem-resistant *Klebsiella pneumoniae* ST258 at a tertiary care hospital over 8 years. MBio.

[22] Wallden K, Rivera-Calzada A, Waksman G (2010). Type IV secretion systems: Versatility and diversity in function. Cell Microbiol..

[23] Krawczun N (2020). Boosting toxic protein biosynthesis: Transient in vivo inactivation of engineered bacterial alkaline phosphatase. Microb. Cell Fact..

[24] Dell'Annunziata F (2020). Outer membrane vesicles derived from *Klebsiella pneumoniae* influence the miRNA Expression profile in human bronchial epithelial BEAS-2B cells. Microorganisms..

[25] Nieckarz M (2017). The role of OmpR in the expression of genes of the KdgR regulon involved in the uptake and depolymerization of oligogalacturonides in *Yersinia enterocolitica*. Front. Cell Infect. Microbiol..

[26] Lee M (2019). Network integrative genomic and transcriptomic analysis of carbapenem-resistant *Klebsiella pneumoniae* strains identifies genes for antibiotic resistance and virulence. mSystems.

[27] Michaelis S, Chapon C, D'Enfert C, Pugsley AP, Schwartz M (1985). Characterization and expression of the structural gene for pullulanase, a maltose-inducible secreted protein of *Klebsiella pneumoniae*. J. Bacteriol..

[28] Boos W, Shuman H (1998). Maltose/maltodextrin system of *Escherichia coli*: Transport, metabolism, and regulation. Microbiol. Mol. Biol. Rev..

[29] Samant S (2008). Nucleotide biosynthesis is critical for growth of bacteria in human blood. PLoS Pathog..

[30] Marti R (2017). Draft genome sequence of *Klebsiella pneumoniae* 704SK6, an OXA-48- and CTX-M-15-encoding wastewater isolate. Genome Announc..

[31] Liu P (2012). Complete genome sequence of *Klebsiella pneumoniae* subsp. *pneumoniae* HS11286, a multidrug-resistant strain isolated from human sputum. J. Bacteriol..

[32] Elemam A, Rahimian J, Mandell W (2009). Infection with panresistant K*lebsiella pneumoniae*: A report of 2 cases and a brief review of the literature. Clin. Infect. Dis..

[33] World Health Organization. *Antimicrobial Resistance*. https://www.who.int/news-room/fact-sheets/detail/antimicrobial-resistance. Accessed 01 Dec 2021 (2020).

[34] Spencer MD (2019). Whole genome sequencing detects inter-facility transmission of carbapenem-resistant *Klebsiella pneumoniae*. J. Infect..

[35] Seki LM (2011). Molecular epidemiology of KPC-2- producing *Klebsiella pneumoniae* isolates in Brazil: The predominance of sequence type 437. Diagn. Microbiol. Infect. Dis..

[36] Snitkin ES (2012). Tracking a hospital outbreak of carbapenem-resistant *Klebsiella pneumoniae* with whole-genome sequencing. Sci. Transl. Med..

[37] Young TM (2020). Animal model to study *Klebsiella pneumoniae* gastrointestinal colonization and host-to-host transmission. Infect. Immunol..

[38] Khurana S, Malhotra R, Mathur P (2020). Evaluation of Vitek(R)2 performance for colistin susceptibility testing for Gram-negative isolates. JAC Antimicrob. Resist..

[39] Ferrari C (2019). Multiple *Klebsiella pneumoniae* KPC clones contribute to an extended hospital outbreak. Front. Microbiol..

[40] David S (2019). Epidemic of carbapenem-resistant *Klebsiella pneumoniae* in Europe is driven by nosocomial spread. Nat. Microbiol..

[41] Bonnin RA (2020). Emergence of new non-clonal group 258 high-risk clones among *Klebsiella pneumoniae* carbapenemase-producing *K. pneumoniae* isolates, France. Emerg. Infect. Dis..

[42] Ondov BD (2016). Mash: Fast genome and metagenome distance estimation using MinHash. Genome Biol..

[43] Seara N (2015). Interhospital spread of NDM-7-producing *Klebsiella pneumoniae* belonging to ST437 in Spain. Int. J. Antimicrob. Agents.

[44] Risan MH, Al-Wattar WM (2017). Molecular Identification of the enterotoxin B gene from *Staphylococcus aureus* and the enterobactin B gene from *Klebsiella pneumoniae*. J. Pharmacogn. Phytochem..

[45] Strakova N, Korena K, Karpiskova R (2021). *Klebsiella pneumoniae* producing bacterial toxin colibactin as a risk of colorectal cancer development—A systematic review. Toxicon.

[46] Martin RM, Bachman MA (2018). Colonization, infection, and the accessory genome of *Klebsiella pneumoniae*. Front. Cell Infect. Microbiol..

[47] Tian X (2019). Molecular epidemiology of and risk factors for extensively drug-resistant *Klebsiella pneumoniae* infections in southwestern China: A retrospective study. Front. Pharmacol..

[48] Li Z, Cao Y, Yi L, Liu JH, Yang Q (2019). Emergent polymyxin resistance: End of an era?. Open Forum Infect. Dis..

[49] Guo C (2015). MLST-based inference of genetic diversity and population structure of clinical *Klebsiella pneumoniae*, China. Sci. Rep..

[50] Ruppe E (2020). From genotype to antibiotic susceptibility phenotype in the order Enterobacterales: A clinical perspective. Clin. Microbiol. Infect..

[51] Ruppe E, Cherkaoui A, Lazarevic V, Emonet S, Schrenzel J (2017). Establishing genotype-to-phenotype relationships in bacteria causing hospital-acquired pneumonia: A prelude to the application of clinical metagenomics. Antibiotics (Basel)..

[52] Lam MMC (2021). A genomic surveillance framework and genotyping tool for *Klebsiella pneumoniae* and its related species complex. Nat. Commun..

[53] Lam MMC, Wick RR, Wyres KL, Holt KE (2020). Genomic surveillance framework and global population structure for *Klebsiella pneumoniae*. bioRxiv.

[54] Leski TA (2013). Identification of blaOXA-(5)(1)-like, blaOXA-(5)(8), blaDIM-(1), and blaVIM carbapenemase genes in hospital *Enterobacteriaceae* isolates from Sierra Leone. J. Clin. Microbiol..

[55] El-Badawy MF, El-Far SW, Althobaiti SS, Abou-Elazm FI, Shohayeb MM (2020). The first Egyptian report showing the co-existence of bla NDM-25, bla OXA-23, bla OXA-181, and bla GES-1 among carbapenem-resistant *K. pneumoniae* clinical isolates genotyped by BOX-PCR. Infect. Drug Resist..

[56] Olaitan AO, Morand S, Rolain JM (2014). Mechanisms of polymyxin resistance: acquired and intrinsic resistance in bacteria. Front. Microbiol..

[57] Poirel L (2015). The *mgr*B gene as a key target for acquired resistance to colistin in *Klebsiella pneumoniae*. J. Antimicrob. Chemother..

[58] Park Y, Choi Q, Kwon GC, Koo SH (2020). Molecular epidemiology and mechanisms of tigecycline resistance in carbapenem-resistant *Klebsiella pneumoniae* isolates. J. Clin. Lab. Anal..

[59] Zhang Q, Lin L, Pan Y, Chen J (2021). Characterization of tigecycline-heteroresistant *Klebsiella pneumoniae* clinical isolates from a chinese tertiary care teaching hospital. Front. Microbiol..

[60] Kelley LA, Mezulis S, Yates CM, Wass MN, Sternberg MJ (2015). The phyre2 web portal for protein modeling, prediction and analysis. Nat. Protoc..

[61] Lam MMC (2018). Tracking key virulence loci encoding aerobactin and salmochelin siderophore synthesis in *Klebsiella pneumoniae*. Genome Med..

[62] Holt KE (2015). Genomic analysis of diversity, population structure, virulence, and antimicrobial resistance in *Klebsiella pneumoniae*, an urgent threat to public health. Proc. Natl. Acad. Sci. USA.

[63] Lam MMC (2018). Genetic diversity, mobilisation and spread of the yersiniabactin-encoding mobile element ICEKp in *Klebsiella pneumoniae* populations. Microb. Genome.

[64] Gu D (2018). A fatal outbreak of ST11 carbapenem-resistant hypervirulent *Klebsiella pneumoniae* in a Chinese hospital: A molecular epidemiological study. Lancet Infect. Dis..

[65] Lam MMC (2018). Population genomics of hypervirulent *Klebsiella pneumoniae* clonal-group 23 reveals early emergence and rapid global dissemination. Nat. Commun..

[66] Faïs T, Delmas J, Barnich N, Bonnet R, Dalmasso G (2018). Colibactin: More than a new bacterial toxin. Toxins..

[67] Gomez-Simmonds A, Uhlemann AC (2017). Clinical implications of genomic adaptation and evolution of carbapenem-resistant *Klebsiella pneumoniae*. J. Infect. Dis..

[68] Tam VH, Schilling AN, Nikolaou M (2005). Modelling time-kill studies to discern the pharmacodynamics of meropenem. J. Antimicrob. Chemother..

[69] Yu L (2019). Synergetic effects of combined treatment of colistin with meropenem or amikacin on carbapenem-resistant *Klebsiella pneumoniae* in vitro. Front. Cell Infect. Microbiol..

[70] Sharma R (2017). Polymyxin B in combination with meropenem against carbapenemase-producing *Klebsiella pneumoniae*: Pharmacodynamics and morphological changes. Int. J. Antimicrob. Agents.

[71] Smith, J.R., Raut, A., Hallesy, J., Aboutaleb, M., & Rybak, M.J. The combination of colistin, meropenem, and daptomycin provides synergistic activity against *Acinetobacter baumannii*. in *25th ECCMID* (Copenhagen, 2015).

[72] Pogue JM, Bonomo RA, Kaye KS (2019). Ceftazidime/avibactam, meropenem/vaborbactam, or both? Clinical and formulary considerations. Clin. Infect. Dis..

[73] Bassetti M, Peghin M, Vena A, Giacobbe DR (2019). Treatment of infections due to MDR gram-negative bacteria. Front. Med. (Lausanne).

[74] Karaiskos I, Lagou S, Pontikis K, Rapti V, Poulakou G (2019). The, "old" and the "new" antibiotics for MDR Gram-negative pathogens: For whom, when, and how. Front. Public Health.

[75] Vena A (2020). Clinical experience with ceftazidime-avibactam for the treatment of infections due to multidrug-resistant gram-negative bacteria other than carbapenem-resistant enterobacterales. Antibiotics (Basel).

[76] Kontopoulou K (2010). Hospital outbreak caused by *Klebsiella pneumoniae* producing KPC-2 beta-lactamase resistant to colistin. J. Hosp. Infect..

[77] Stone SP (2007). The ORION statement: Guidelines for transparent reporting of outbreak reports and intervention studies of nosocomial infection. Lancet Infect. Dis..

[78] Tacconelli E (2018). Surveillance for control of antimicrobial resistance. Lancet Infect. Dis..

[79] ANVISA. Agencia Nacional de Vigilância Sanitária (Nota Técnica No. 01/2013). *Medidas de Prevenção e Controle de Infecções por Enterobactérias Multirresistentes*. http:// portal.anvisa.gov.br/wps/wcm/connect/ea4d4c004f4ec3b98925d9d785749fbd/ Micro soft+Word++NOTA+T%C3%89CNICA+ENTEROBACTERIAS+17+04+2013%281%29.pdf? MOD=AJPERES. Accessed 16 June 2018 (2013).

[80] Nordmann P, Poirel L, Dortet L (2012). Rapid detection of carbapenemase-producing *Enterobacteriaceae*. Emerg. Infect. Dis..

[81] Song W (2015). Combined use of the modified Hodge test and carbapenemase inhibition test for detection of carbapenemase-producing *Enterobacteriaceae* and metallo-beta-lactamase-producing *Pseudomonas* spp.. Ann. Lab. Med..

[82] CLSI. *Twenty-Sixth Informational Supplement. CLSI Document M100-S26*. (Clinical and Laboratory Standards Institute, 2016). Accessed 16 June 2017.

[83] EUCAST. *European Committee on Antimicrobial Susceptibility Testing Breakpoint Tables for Interpretation of MICs and Zone Diameters*. Version 6.0, valid from 2016-01-01. http://www.eucast.org/clinical_breakpoints/. Accessed 16 June 2016.

[84] Hansen DS, Skov R, Benedi JV, Sperling V, Kolmos HJ (2002). *Klebsiella* typing: Pulsed-field gel electrophoresis (PFGE) in comparison with O:K-serotyping. Clin. Microbiol. Infect. Dis..

[85] Diancourt L, Passet V, Verhoef J, Grimont PA, Brisse S (2005). Multilocus sequence typing of *Klebsiella pneumoniae* nosocomial isolates. J. Clin. Microbiol..

[86] Coil D, Jospin G, Darling AE (2015). A5-miseq: An updated pipeline to assemble microbial genomes from Illumina MiSeq data. Bioinformatics.

[87] Seemann T (2014). Prokka: Rapid prokaryotic genome annotation. Bioinformatics.

[88] Page AJ (2015). Roary: Rapid large-scale prokaryote pan genome analysis. Bioinformatics.

[89] Larsen MV (2012). Multilocus sequence typing of total-genome-sequenced bacteria. J. Clin. Microbiol..

[90] Kumar S, Stecher G, Li M, Knyaz C, Tamura K (2018). MEGA X: Molecular evolutionary genetics analysis across computing platforms. Mol. Biol. Evol..

[91] Saitou N, Nei M (1987). The neighbor-joining method: A new method for reconstructing phylogenetic trees. Mol Biol Evol.

[92] Tamura K, Nei M, Kumar S (2004). Prospects for inferring very large phylogenies by using the neighbor-joining method. Proc. Natl. Acad. Sci. USA.

[93] Feldgarden M (2019). Validating the AMRfinder tool and resistance gene database by using antimicrobial resistance genotype-phenotype correlations in a collection of isolates. Antimicrob. Agents Chemother..

[94] Jia B (2017). CARD 2017: Expansion and model-centric curation of the comprehensive antibiotic resistance database. Nucleic Acids Res..

[95] Zankari E (2012). Identification of acquired antimicrobial resistance genes. J. Antimicrob. Chemother..

[96] Gupta SK (2014). ARG-ANNOT, a new bioinformatic tool to discover antibiotic resistance genes in bacterial genomes. Antimicrob. Agents Chemother..

[97] Chen L, Zheng D, Liu B, Yang J, Jin Q (2016). VFDB 2016: Hierarchical and refined dataset for big data analysis–10 years on. Nucleic Acids Res..

[98] Carattoli A (2014). In silico detection and typing of plasmids using PlasmidFinder and plasmid multilocus sequence typing. Antimicrob. Agents Chemother..

[99] Ingle DJ (2016). In silico serotyping of *Escherichia coli* from short read data identifies limited novel O-loci but extensive diversity of O:H serotype combinations within and between pathogenic lineages. Microb. Genome.

[100] Doster E (2020). MEGARes 2.0: A database for classification of antimicrobial drug, biocide and metal resistance determinants in metagenomic sequence data. Nucleic Acids Res..

[101] Schwengers O (2020). Platon: Identification and characterization of bacterial plasmid contigs in short-read draft assemblies exploiting protein sequence-based replicon distribution scores. Microb. Genome..

[102] Poirel L, Jayol A, Nordmann P (2017). Polymyxins: Antibacterial activity, susceptibility testing, and resistance mechanisms encoded by plasmids or chromosomes. Clin. Microbiol. Rev..

[103] Altschul SF (1997). Gapped BLAST and PSI-BLAST: A new generation of protein database search programs. Nucleic Acids Res..

[104] Yang X, Ye L, Chan EW, Zhang R, Chen S (2021). Characterization of an IncFIB/IncHI1B plasmid encoding efflux pump TMexCD1-TOprJ1 in a clinical tigecycline- and carbapenem-resistant *Klebsiella pneumoniae* strain. Antimicrob Agents Chemother..

[105] CLSI. *Thirty Informational Supplement. CLSI Document M100-S30th*. (Clinical and Laboratory Standards Institute, 2020). Accessed 10 Jan 2020.

[106] Mikhail S (2019). Evaluation of the synergy of ceftazidime-avibactam in combination with meropenem, amikacin, aztreonam, colistin, or fosfomycin against well-characterized multidrug-resistant *Klebsiella pneumoniae* and *Pseudomonas aeruginosa*. Antimicrob. Agents Chemother..

[107] Kebriaei R (2020). Mechanistic insights into the differential efficacy of daptomycin plus beta-lactam combinations against daptomycin-resistant *Enterococcus faecium*. J. Infect. Dis..

