# Supplementary material for: Outbreak report of polymyxin-carbapenem-resistant Klebsiella pneumoniae causing untreatable infections evidenced by synergy tests and bacterial genomes
Source: Sci Rep. 2023 Apr 17;13:6238. doi: 10.1038/s41598-023-31901-4 (PMC10110528; doi:10.1038/s41598-023-31901-4)
Supplement: Supplementary file 2 — Supplementary Information. [file 41598_2023_31901_MOESM2_ESM.pdf]

[Supplementary Data]

Outbreak report of polymyxin-carbapenem-resistant *Klebsiella pneumoniae* causing untreatable infections evidenced by synergy tests and bacterial genomes

Marisa Zenaide Ribeiro Gomes[1,2,3*], Elisangela Martins de Lima[2], Caio Augusto Martins Aires[3**], Polyana Silva Pereira[3], Juwon Yim[4], Fernando Henrique Silva[1], Caio Augusto Santos Rodrigues[2], Thamirys Rachel Tavares e Oliveira [3], Priscila Pinho da Silva[1], Cristiane Monteiro Eller[1], Claudio Marcos Rocha de Souza[3], Michael J. Rybak[4], Rodolpho Mattos Albano[5], Antonio Basílio de Miranda[1], Edson Machado[1***], Marcos Catanho[1], and Nucleus of Hospital Research (NPH) study collaborators

[1]Laboratório de Genética Molecular de Microrganismos, Instituto Oswaldo Cruz, Fundação Oswaldo Cruz, Rio de Janeiro, Brazil; [2]Hospital Federal Servidores do Estado, Ministry of Health, Rio de Janeiro, Brazil; [3]Laboratório de Pesquisa em Infecção Hospitalar, Instituto Oswaldo Cruz, Fundação Oswaldo Cruz, Rio de Janeiro, Brazil; [4]Anti-Infective Research Laboratory, Eugene Applebaum College of Pharmacy and Health Sciences, Department of Medicine, Division of Infectious Diseases, School of Medicine, Wayne State University, Detroit, Michigan, USA; [5]Departamento de Bioquímica, IBRAG, Universidade do Estado do Rio de Janeiro, Rio de Janeiro, Brazil

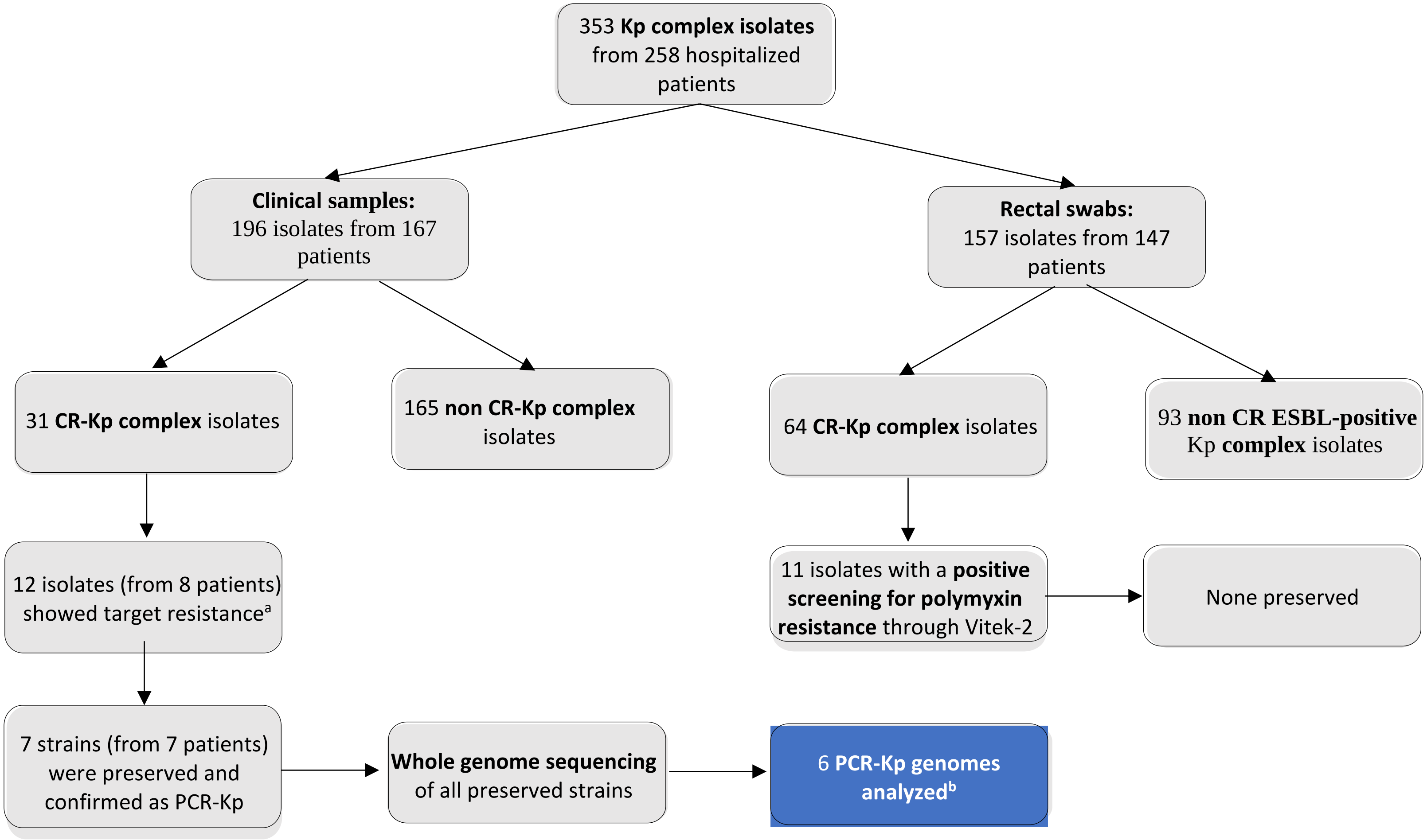
353 Kp complex isolates from 258 hospitalized patients
Clinical samples: 196 isolates from 167 patients
Rectal swabs: 157 isolates from 147 patients
31 CR-Kp complex isolates
165 non CR-Kp complex isolates
64 CR-Kp complex isolates
93 non CR ESBL-positive Kp complex isolates
12 isolates (from 8 patients) showed target resistance[a]
11 isolates with a positive screening for polymyxin resistance through Vitek-2
None preserved
7 strains (from 7 patients) were preserved and confirmed as PCR-Kp
Whole genome sequencing of all preserved strains
6 PCR-Kp genomes analyzed[b]

Algorithm 1. *K. pneumoniae* (Kp) complex isolates, without repetition, according to clinical and surveillance samples and resistance profile to any carbapenems and polymyxins, tertiary federal hospital, December 2014 to August 2015. [a]Target resistance corresponds to intermediate/resistance profile to any carbapenems (meropenem, imipenem or ertapenem) screening positive for polymyxins resistance. [b] One strain (CCBH19496) was excluded from the genome analysis due to experimental problems. CR-Kp, carbapenem-resistant Kp; ESBL, extended spectrum beta-lactamase; PCR-Kp, polymyxin-carbapenem-resistant *K. pneumoniae*

Supplementary Table 1. Demographic and clinical characteristics of patients infected by polymyxin-carbapenem-resistant *K. pneumoniae*[a]

Case #[b]	Gender	Age	Previous Comorbidity	Clinical Sample	Type/source of infection	CCBH Strain	MLST
1[c]	Male	72	Chronic renal failure, admitted with Tetanus, developed CR-*Acinetobacter baumannii* and CR-*Pseudomonas aeruginosa* VAP, Nosocomial diarrhea[e]	Blood	Sepsis/CRBSI	17440	ST 437
2	Female	25	Previous healthy, admitted with Rhombencephalitis, developed *Stenotrophomonas maltophilia* VAP, **13 days of previous rectal colonization with colistin-carbapenem-resistant *K. pneumoniae***, Nosocomial diarrhea[e], Acute renal failure, Hemodialysis	Tracheal aspirate	Sepsis/VAP (>10^6 UFC in tracheal aspirate)	17428	ST 437
3	Male	82	Multiple myeloma, **13 days of previous rectal colonization with colistin-carbapenem-resistant *K. pneumoniae*** and developed Nosocomial diarrhea	Blood	Polymicrobial sepsis[f]/UTI	17724	ST 11
4[d]	Male	55	Diabetes mellitus, Chronic renal failure, Peritoneal dialysis, Occlusive peripheral arterial disease, admitted for Femorofibular bypass	Abscess	SSI	19496	ST 437
5[c]	Male	79	Myeloid acute leukemia, Febrile neutropenia, Healthcare-associated pneumonia and Septic shock, Massive bronco aspiration	BAL	Sepsis/polymicrobial VAP (>10^6 CFU both agents in BAL)[g]	NP[a]	-
6	Female	85	Renal abscess, admitted for Nephrectomy, developed Acute renal failure requiring Hemodialysis, Deep vein thrombosis, previous VRE colonization, **56 days of previous rectal colonization with colistin-carbapenem-resistant *K. pneumoniae***, Pneumonia and Pleural effusion, Nosocomial diarrhea	Urine	Sepsis/UTI (>10^5 CFU in urine)	19867	ST 437
7[d]	Male	65	Hypertension, Diabetes mellitus, Chronic renal failure, Rheumatoid arthritis	Urine	UTI (>10^5 CFU in urine)	19868	ST 11
8	Female	70	Hypertension, Chronic atrial fibrillation, Diabetes mellitus, Occlusive peripheral arterial disease, admitted for Femorofibular bypass and transmetatarsal amputation, developed Surgical site infection, MRSA, VRE and **CRE (93 and 122 days before) colonization**, MS *Proteus mirabilis* UTI, Bronchoaspiration, Acute exacerbation of Chronic renal failure, Hemodialysis, Septic shock, Nosocomial diarrhea, Infected decubitus ulcer and Osteomyelitis	Urine	Urinary-tract infection (>10^5 CFU in urine) evolving to possible XDR CR-Kp UTI secondary sepsis in 25 days	19771	ST 437

CR, carbapenem-resistant; CRE, carbapenem-resistant Enterobacteriaceae; MDR, multidrug-resistant; MIC, minimum inhibitory concentration; MLST, multilocus sequence typing; MRSA; methicillin-resistant *Staphylococcus aureus*; MS, multi-susceptible; NP, not preserved; UTI, urinary tract infection; VAP, ventilator-associated pneumonia; VRE, vancomycin-resistant *Enterococcus*; Cases reported in order of strains detection; [a]Including non preserved CR-Kp screening positive for polymyxin resistance. [b]Reported cases in order of detection of target isolates. [c]Rectal swab negative for CRE previously or during infection. [d]Did not perform surveillance rectal swabs during hospitalization; [e]Treated empirically *for Clostridium difficile* associated diarrhea; [f]Polymicrobial infection with *Enterococcus faecium* susceptible to vancomycin; [g]Polymicrobial infection with possible XDR *Acinetobacter baumannii*;

Complete Report of Index Cases:

Case 1 (index case)**.** A 72-year-old 80 Kg white male, chronic renal failure previously managed with conservative treatment was recovering from severe accidental tetanus, suspected *Clostridium difficile* associated diarrhea (CDAD), and hospital-acquired ventilator-associated pneumonia (VAP) caused by carbapenem-resistant (CR) *Acinetobacter baumannii* and CR *Pseudomonas aeruginosa*, when he was diagnosed with sepsis evolving to septic shock on day 39 of hospitalization. The etiology of bloodstream infection was initially classified as PDR *K. pneumoniae* (CCBH17440). Previously to this diagnosis and during his hospitalization at the infectious diseases intensive care unit (ICU), the patient received several antimicrobials to treat hospital infections which included polymyxin B (28 days), tigecycline (15 days), meropenem (11 days), cefepime (11 days), metronidazole (10 days), teicoplanin (8 days), amikacin (three days), caspofungin (three days) and sulfamethoxazole-trimetoprim (two days). He died on day 43 after treatment with polymyxin B (1 million IU q12h) and 3-hours-infusion meropenem (2g q12h) for four days, gentamicin (160mg q72h) for three days, and teicoplanin (400mg IV q12h) for one day, with dosages based on renal clearance. Appendix Table 1 shows patient and CCBH17440 strain data profiles.

Case 2. A previously healthy 25-year-old 85Kg black female with meningoencephalitis, later diagnosed as rhombencephalitis, was admitted to the same bed as the index case on the fifth day after his death. This bed was cleaned regularly and remained unoccupied for four days before this patient occupied it. Ceftriaxone, vancomycin, acyclovir/ganciclovir and later a course of ampicillin was given to this patient to treat her baseline disease. She also received pulse therapy with methylprednisolone (1g IV q24h) for three days but did not respond to the treatment. During the hospital course, she had worsened level of consciousness requiring mechanical

ventilation. Four days later, she initiated empirical therapy for VAP with meropenem, polymyxin B, and linezolid for 21 days. On the 21st day of admission, she was transferred to adult medical-surgical ICU at the same hospital because she developed acute renal failure requiring hemodialysis. Rectal and tracheal colonization with possible XDR and possible PDR *K. pneumoniae* strains were detected eight and 18 days later, respectively. Further, she received trimethoprim-sulfamethoxazole to treat *Stenotrophomonas maltophilia* VAP for 12 days, followed by another course of antibiotics comprising polymyxin B, meropenem, amikacin, vancomycin, and caspofungin for clinical sepsis and empirical therapy for CDAD, evolving with copious diarrhea until her death. PDR *K. pneumoniae* strains were isolated again from urine ($\geq 10^5$ CFU) and tracheal aspirate ($\geq 10^6$ CFU) (CCBH17428), causing sepsis due to VAP, without response to the combination of polymyxin B (1 million IU q12h), amikacin (1g q24h), vancomycin (1g q12h) and amphotericin B lipid complex (300mg q24) for nine days and levofloxacin (750mg IV q24h) for one day.

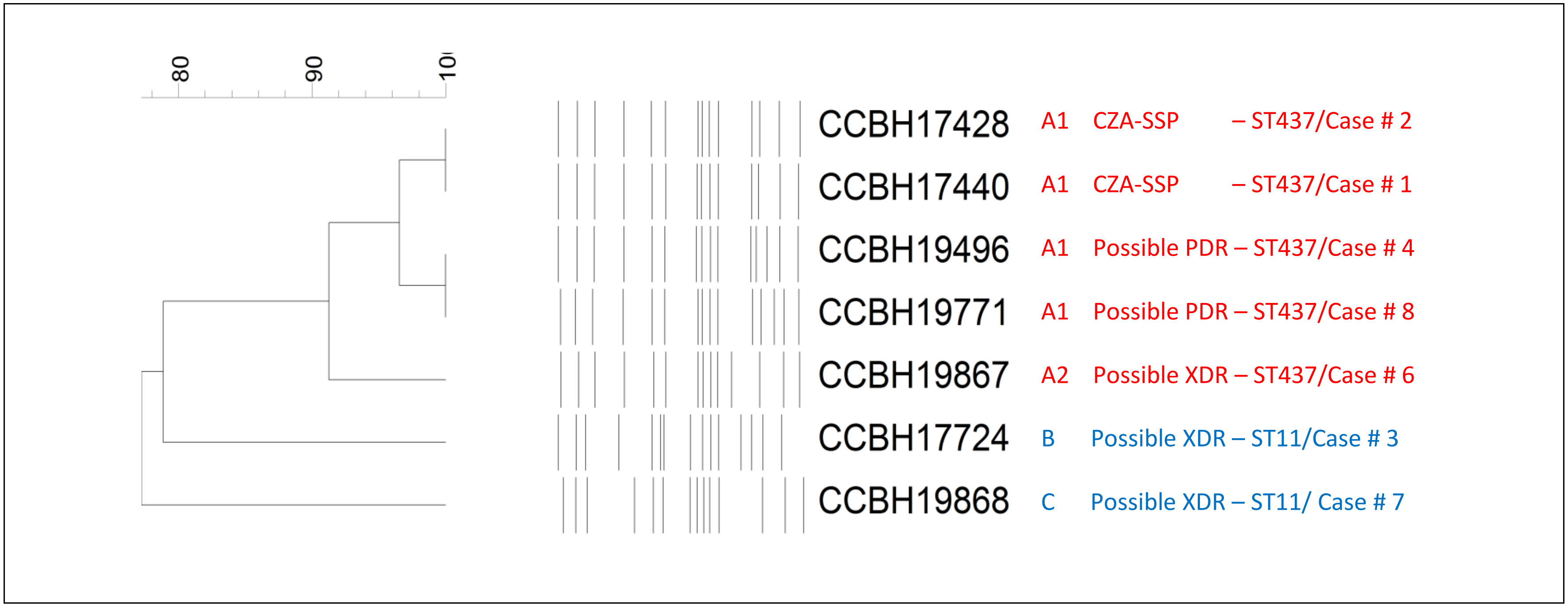

Supplementary Figure 1. Pulsed-field gel electrophoresis patterns and dendrogram of XbaI-digested genomic DNA of ST437 (red) and ST11 (blue) polymyxin-carbapenem-resistant *Klebsiella pneumoniae* (PCR-Kp) isolates. CZA-SSP, ceftazidime-avibactam single susceptible profile; PDR, pandrug-resistant; XDR, extensively-drug resistant. Case number in order of detection of PCR-Kp isolates.

Supplementary Table 2. Antimicrobial susceptibility profile of clinical polymyxin-carbapenem-resistant *K. pneumoniae* isolates by multilocus sequence typing (MLST) and not preserved carbapenem-resistant *K. pneumoniae* complex isolates screened positive for polymyxin resistance

Strain #/Clinical sample	CCBH Strain/ Case #	MLST	Antimicrobial Susceptibility Testing[a] MIC (mg/L)																							
			AKN	GEN	NET	TOB	SAM	PTZ	CAZ	CTT	CZ	CZA	FEP	FOX	XM	ERT	IMP	MEM	CIP	LEV	SUT	TCG	FOS	COL	PB	DOXY
**1/blood**	**17440/1**	**ST437**	>64	>64	>64	>64	≥32	≥128	16	>64	>64	**0.5 (S)**	≥64	≥64	>256	64	≥32	>64	≥4	>64	>64	2	>64	32	24	8
**2/urine**	**NP/2**	**NT**	≥64	≥16	NT	NT	NT	≥128	NT	NT	NT	NT	≥64	NT	≥64	≥8	NT	≥16	≥4	NT	≥320	2	NT	NT	R	NT
**3/tracheal aspirate**	**17428/2**	**ST437**	≥64	≥64	≥64	>64	≥32	≥128	16	≥64	≥64	**0.5 (S)**	≥64	≥64	>256	64	≥32	>64	≥4	>64	>64	2	>64	64	32	8
**4/tracheal aspirate**	**NP/2**	**NT**	≥64	≥16	NT	≥16	≥32	≥128	16	NT	NT	NT	≥64	≥64	≥64	≥8	≥16	≥16	≥4	≥8	R	2	NT	≥16	NT	NT
**5/blood**	**17724/3**	**ST11**	16 (S-CLSI) (NS-EUCAST)	**≤1 (S)**	NT	NT	≥32	≥128	16	NT	NT	NT	4	32	≥64	≥8	≥16	≥16	≥4	NT	NT	**1 (S)**	NT	≥16	32	NT
**6/abscess secretion**	**NP/4**	**NT**	≥64	**2 (S)**	NT	NT	NT	≥128	NT	NT	NT	NT	≥64	NT	≥64	≥8	≥4	≥16	≥4	NT	≥320	**2**	NT	NT	32	NT
**7/abscess liquid**	**19496/4**	**ST437**	≥64	≥16	NT	NT	≥32	≥128	16	NT	NT	NT	≥64	≥64	≥256	>32	≥16	>32	≥4	≥8	R	1.5 (NS)	NT	8	8	NT
**8/tracheal aspirate**	**NP/5**	**NT**	≥64	≥16	NT	NT	≥32	≥128	16	NT	NT	NT	≥64	≥64	≥64	≥8	≥16	≥16	≥4	NT	NT	1.5 (NS)	NT	≥16	NT	NT
**9/urine**	**19867/6**	**ST437**	**4 (S)**	NT	NT	NT	NT	≥128	≥16	NT	NT	NT	≥64	NT	≥64	≥8	≥4	≥16	1 (S-CLSI) (NS-EUCAST)	NT	160	2	NT	NT	24	NT
**10/urine**	**NP/6**	**NT**	**4 (S)**	NT	NT	NT	NT	≥128	≥16	NT	NT	NT	≥64	NT	≥64	≥8	≥4	≥16	1 (S-CLSI) (NS-EUCAST)	NT	160	2	NT	NT	24	NT

**11/urine**	**19868/7**	**ST11**	16 (S-CLSI) (NS-EUCAST	**≤1 (S)**	NT	NT	NT	≥128	≥16	NT	NT	NT	NT	NT	≥64	4	≥4	≥16	≥4	NT	≥320	NT	NT	NT	24	NT
**12/urine**	**19771/8**	**ST437**	≥64	≥16	NT	NT	NT	≥128	NT	NT	NT	NT	≥64	NT	≥64	≥8	NT	>16	≥4	NT	≥320	2	NT	NT	12	NT

MIC, minimum inhibitory concentration; NP, not preserved; NS, non-susceptible; NT, not tested; R, resistant; S, susceptible. [a] Broth microdilution in all preserved strains for amikacin (AKN), ampicillin-sulbactam (SAM), cefazolin (CZ), cefepime (FEP), cefotetan (CTT), cefoxitin (FOX), ceftazidime (CAZ), cefuroxime (XM), ciprofloxacin (CIP), colistin (COL), doxycycline (DOXY), ertapenem (ERT), fosfomycin (FOS), gentamicin (GEN), levofloxacin (LEV), meropenem (MEM), netilmicin (NET), piperacillin-tazobactam (PTZ), trimethoprim-sulfamethoxazole (SUT) and tobramycin (TOB), according to CLSI 2016 criteria[1] and/or EUCAST 2016 breakpoints[2]. Etest methods (Biomérieux) performed for TGC, imipenem (IMP), polymyxin B (PB) and ceftazidime-avibactam (CZA). We reported CLSI and EUCAST breakpoint interpretations in the Table whenever they have any difference. Antimicrobial susceptibility tests in non-preserved strains based in routine laboratory with Vitek 2 system. Preserved strains displaying non-susceptibility by disc diffusion (Oxoid; Hampshire, UK) are shown in parenthesis as following (not in the Table): amoxicillin-clavulanate (n=6 tested: 17440, 17428, 19496, 19867, 19868, 19771), aztreonam (n=3 tested: 17440, 17428, 19496), cefotaxime (n=3 tested: 17440, 17428, 19496), ceftaroline (n=3 tested: 17440, 17428, 19496), cephalothin (n=6 tested: 17440, 17428, 19496, 19867, 19868, 19771), chloramphenicol (n=3 tested: 17440, 17428, 19496), doripenem (n=3 tested: 17440, 17428, 19496), minocycline (n=3 tested: 17440, 17428; CCBH19496 showing susceptibility), tetracycline (n=3 tested: 17440, 17428; CCBH19496 showing susceptibility) and ticarcillin-clavulanate (n=3 tested: 17440, 17428, 19496), according to CLSI criteria[1]. All tests in duplicate (except

in not preserved strains), using quality control strains: *Staphylococcus aureus* ATCC® 29213, *Pseudomonas aeruginosa* ATCC® 27853, *Escherichia coli* ATCC® 25922 and *K. pneumoniae* ATCC® 700603 (for ceftazidime-avibactam)[1]. Light gray cells show the susceptible MIC. Isolates reported in order of detection.

Supplementary Table 3. Genome *de novo* assembly statistics of clinical ST437 and ST11 polymyxin-carbapenem-resistant *K. pneumoniae* samples.

Features	Strains/Case #					
	CCBH17440/1	CCBH17428/2	CCBH17724/3	CCBH19867/6	CCBH19868/7	CCBH19771/8
MLST	ST437	ST437	ST11	ST437	ST11	ST437
Reads	2,881,022	4,839,746	2,161,468	6,015,334	4,909,048	5,177,646
Base-pairs	5,608,888	5,616,266	5,571,467	5,815,502	5,916,628	5,501,114
N50 (bp)	204,267	204,143	219,649	178,538	205,883	174,955
Contigs	85	105	74	127	215	88
Coverage (%)	154.0	165.01	65.21	200.82	163.7	173.0
GC (%)	57	57	57	57	57	57
Genes	5,395	5,396	5,379	5,630	5,655	5,255
CDS	5,298	5,298	5,280	5,533	5,556	5,159
tRNA	84	84	84	84	84	84
rRNA	12	13	14	12	14	11
GenBank Assembly Accession[a]	GCA_001715215.1	GCA_017565915.1	GCA_017565865.1	GCA_017565945.1	GCA_017566015.1	GCA_017565885.1

[a]BioProject accessions PRJNA336378 (CCBH17440) and PRJNA678746 (other strains); CDS, protein-coding sequences, GC, guanine-cytosine content; rRNA, ribosomal RNA; tRNA, transfer RNA; ST437 (red) and ST11 (blue). ST437 CCBH19496 (case 4)

had contaminated genomic sequences and was excluded from the analysis. CR-Kp screened positive for polymyxin from case 5 was not preserved. Case number in order of strains detection.

Supplementary Table 4. Antimicrobial resistance profile and related genes in clinical ST437 (red) and ST11 (blue) polymyxin-carbapenem-resistant *K. pneumoniae* samples.

AMR profile and related genes[a]		CCBH Strains/Case # MLST					
		17440/1	17428/2	17724/3	19867/6	19868/7	19771/8
		ST437	ST437	ST11	ST437	ST11	ST437
**Resistance score[b]**		3	3	3[c]	3	3	3
**Drug class resistance**		**Presence of AMR related Genes[d]**					
**Aminoglycosides (AGly)**	***aac(6')Ib-cr/acrA/acrB/kpnE/kpnF/kpnG/kpnH***	■	■	■	■	■	■
	armA	■	■		■		■
	aadA2	■		■		■	
	aph(3'')-Ia					■	
**Fluoroquinolones (Flq)**	***aac(6')Ib-cr/gyrA-83/ kpnG/kpnH/oqxA/oqxB/parC-80***	■	■	■	■	■	■
	qnrA1	■	■		■		
**Beta-lactams (Bla)**	***ampH/bla$_{CTX-M-97}$/bla$_{OXA-1}$/bla$_{SHV11}$/ bla$_{SHV}$35Q/kpnE/kpnF/kpnG/ kpnH***	■	■	■	■	■	■
	bla$_{KPC-2}$	■	■		■	■	■
	ompK36GD	■	■		■		■
	bla$_{CTX-M-9}$	■	■		■		
	ompK35-24% mutation			■		■	
	bla$_{CTX-M-14}$				■		

	bla_{TEM-1}/bla_{DHA-1}
**Chloramphenicol (Phe)**	***catB4***
	catA1
	catB3
**Trimethoprim (Tmt)**	***dfrA***
	dfrA8
	dfrA12
**Sulfamethoxazole (Sul)**	***sul1***
**Rifampim (Rif)**	***kpnE/kpnF***
	arr-3
**Macrolides (Mls)**	***kpnE/kpnF/kpnG/kpnH***
	***mph(E)/msr*(E)**
	mph(A)
	erm42
**Tetracyclines (Tet)**[e]	***kpnE/kpnF***
	tetA/tetR
**Tigecycline (Tgc)**[f]	Val*130* to Ala mutation in ***oqx*R**
**Fosfomyin (Fos)**	***fos*A**

**Polymyxins (Pol)**	***mgr*B** truncation	49%	49%	62%	49%	62%	49%
	kpnE/kpnF/kpnG/kpnH, and absence of ***pmrB***						

[a]Protein names, predicted resistance and referral literature in Supplementary Table 4A (Excel file 1); [b]Resistance score according to Lam et al. 2020[3]; [c]Positive carbapenemase screening test, negative for *bla*$_{\text{KPC-2}}$, *bla*$_{\text{NDM-1}}$ and *bla*$_{\text{OXA-48}}$; [d]Table cell colored in red (ST437) or blue (ST11) represents the presence of searched genes, in blank means the absence of the genes; [e]Although we did not observe mutations in the genes associated to tetracycline resistance, the lack of knowledge about the expression levels of efflux pump genes detected may have precluded the identification of this resistance mechanism[4,5]. [f]Regarding tigecycline resistance, we did not detect mutation in *ram*R or *rps*J and IS26-like elements flanking *oqx*A and *oqx*B genes in all strains[6]. *tetA* gene was present in CCBH19868 only, without any mutation[7]. The presence of the *tet*X, *tet*L and *tet*M genes, efflux pump *tmex*CD1-*topr*J1 gene cluster and deleterious mutations in *acrA/acrB/acrR/lon/marA/marR/ramA/rarA/soxR/soxS/tolC* genes were not investigated [8-12]. AMR, antimicrobial resistance; MLST, multilocus sequence typing. Case number in order of strain detection.

Supplementary Table 5. Virulence score and associated genes and features according to the main biological characteristics predicted in the literature, possibly leading to specific stages (yellow, pink and green) of ST437 (red) and ST11 (blue) polymyxin-carbapenem-resistant *K. pneumoniae* infection.

Virulence Genes and Features	CCBH Strain/Case # MLST						Main Predicted Biological Characteristics[c]	Stages in bacterial infection possibly related		
	17440/ 1	17428/ 2	17724/ 3	19867/ 6	19868/ 7	19771/ 8				
	ST437	ST437	ST11	ST437	ST11	ST437				
**Virulence Score**[a]	**0**	**0**	**0**	**0**	**2**	**0**				
**Gene**/Locus/Operon/Other features[b,c]								**Adherence and mucosal colonization**	**Invasion and systemic infection**	**Resistance, survival or proliferation**
"string-test" negative, however mucoid aspect[d]			**NE**	**NE**	**NE**	**NE**	Capsule, resistance to phagocytosis by polymorphonuclear leukocytes and killing by complement, antimicrobial peptides and other bactericidal molecules, and abilities to adhere to intestinal or urinary-tract epithelial cells. Increased survival in serum and tissue			
K (capsule) locus, ***wzi***	KL36 wzi 109	KL36 wzi 109	KL27 -	KL36 wzi 109	KL27 -	KL36 wzi 109				
kpnE/kpnF										
uge										
ecpA/ecpB/ecpC/ecpD/ ecpE/ecpR/fimH/fliY/ mrkA/mrkC/mrkD/mrkF/ mrkH/mrkI/mrkJ/yfcO/ yfcR							Fimbrium, surface adhesion, motility, cell-cell interaction, bind host derived matrix component, the ability to colonize and/or biofilm formation, conjugation and DNA uptake. Increases biofilm production and thus contribute to colonization. Adherence to environmental, biological- and nonbiological surfaces.			
wabG/kdsA/msbA/pagP/ msbA/pgi/yrbI							Biosynthesis of the core LPS, endotoxin and immune evasion, promoting bacterial			

O locus	O4	O4	O2	best match = O4	O2	O4	dissemination and colonization of internal organs after the onset of bacteremia, outer membrane stability and protection against the outside environment.			
uge										
gapA/kdsA/mdh/ *ECS88_3547 locus* of ***nlpI*** gene***/ompA/tolQ/tolR/tsf/tsx***							Present in outer membrane vesicles (OMVs). OMVs have a role in colonization niche, transmission of virulence factors into host cells, and modulation of host defense. OMVs function as a shuttle to deliver LPS into the cytosol of the host cell. OMVs derived from *K. pneumoniae* are important secretory nanocomplexes that elicit a potent inflammatory response. *K. pneumoniae* OMVs increased the blood stream dissemination of whole cell bacteria. MDR strains produced 2-3 times more OMVs than their respective antibiotic susceptible strains			
ompA/ompK37/phoE/ycfM/tolC							Outer membrane components (resistance to environmental stress, serum resistance, membrane functionality, cell shape, protectin). Porin activity: its loss, lower expression or the modification of channel size are associated with AMR			
ompK36GD										
ompK35*-24%/****traT***										
acrA/acrB/acrD/ fliY/gltP/kpnE/ kpnF/kpnG/kpnH/oqxA/ oqxB/sugE/yadG/yadH/ yfcA/yfcJ/znuB/znuC							Efflux or transport system, its overexpression is commonly found in multidrug-resistant clinical isolates, resistance to disinfectant, environmental adaptation.			
EC55989_3335 *gene/****secY***							Component of secretion system, role in horizontal gene transfer			
entA/entB/fepC/fhuF/ iroD[e]***/iroN***[e]***/iutA***[f]***/sitA***							Iron acquisition; Iron regulatory proteins are involved in the synthesis of siderophore or iron transport system, promoting bacterial growth, inducing inflammatory cytokines and bacterial dissemination or growth in blood			
yersiniabactin ***ybt17*** locus, ICEKp10/***fyuA/irp1/irp2/ybtA/ybtE/ybtP/ybtQ/ybtS/ybtT/ybtU/ybtX***										
acrR/baeR/cpxA/emrR/ int/marA/marR/oqxR/ phoP/phoQ/phoR/ramA/ ramR/rarA/rfaH/soxR/ soxS							Regulatory system or regulator, involved in transcription od DNA binding. Its overexpression or dysregulation is commonly found in multidrug-resistant clinical isolates, resistance to disinfectant or environmental adaptation			

envR/fis/robA/sdiA	
colibactin cluster[g] ***clbABCDEFGHILMNOPQR***	Toxins, hemolysins, transferase activity, magnesium ion binding, pathway fatty acid biosynthesis, double strand DNA breaks in eukaryotic cells or genotoxic involved in development of colorectal cancer in *E. coli.* Proposed to facilitate mucosal and gut colonization, and dissemination to the blood and other organs. Associated with liver abscess and present in some hypervirulent clones
ureA/ureB/ureD	Urea degradation, nitrogen metabolism, required for efficient bacterial gastrointestinal colonization
kpnE/kpnF	Survival in hyperosmotic conditions in a microaerobic environment such as the upper gastrointestinal tract
Hpt/lplA/lipA	Intracellular survival
***ira*M/*ira*P**	Stress responses in Enterobacteriaceae, cellular response to starvation
tig	Trigger factor, protease, act in cell division and cell cycle
purT	Purine synthesis, essential for cellular replication under conditions in which bacteria are unable to scavenge for exogenous nucleotides
trpS	Tryptophan biosynthesis
argA	L-arginine biosynthesis in *Klebsiella variicola;* senses arginine fluctuations and regulates the virulence of enterohemorrhagic *E. coli*
glnA	Glutamine synthesis, activated by nitrogen deprivation
hemE*/hemF/*hemH/ hemK/hemN	Heme biosynthesis in the process of bacteria obtain iron from heme
fadA	Fatty acid biosynthesis, which is part of lipid metabolism; fatty acid usage as a sole carbon

Irp1/clbB						and energy source during aerobic growth	
rhaA/rhaB/rhaD/rhaR/ rhaS/rhaT						L-rhamnose (L-Rha) metabolism, a deoxy-hexose sugar commonly found in plants as a part of complex pectin polysaccharides and as a common component of bacteria cell wall. Enterobacteriaceae are capable of utilizing L-Rha as a carbon source	
yfcF/yfcG						Detoxify toxic compounds in bacterial cell, biodegradation of xenobiotics and several monocyclic aromatic compounds such as toluene, xylenes, phenols, and atrazine, defense against oxidative stress, protection against chemicals, and resistance to antimicrobial drugs in *Acinetobacter* sp. Involved in *Klebsiella* sp. metabolism of environmental lignin mineralization. Hydrogen peroxide resistance, defense against oxidative stress in *E. coli*	
yfcE						Hydrolyze biomolecules implicated in DNA repair, post-translational modification, biomineralization, energy metabolism, and signal transduction through regulation of secondary metabolites circulation	
kpnE/kpnF[h]						Mutation in the putative efflux genes *kpn*E, *kpn*F described increasing the susceptibility to sodium dodecyl sulfate, deoxycholate, dyes, benzalkonium chloride, chlorhexidine, and triclosan	
kpnG/kpnH[h]						Described insertional inactivation of *kpnGH* increases the susceptibility to dyes and detergents such as ethidium bromide, acriflavine, deoxycholate, sodium dodecyl sulphate, and disinfectants benzalkonium chloride, chlorhexidine and triclosan	
terB/terE						Heavy metals resistance, protection from oxidative stress or agents causing membrane damage. Tellurium resistance is independently associated with disease and with hypervirulence in CG23, CG65 and CG86	
terA/terC/terF/terY2/ terY3							
terD/terX/terW/terZ							

[a]Virulence score according to Lam et al. 2020[3]; [b]red and blue colors mean the presence of the gene(s) respectively in ST437 and ST11 strains, while color in blank represents the absence of the gene(s). [c]Genes, protein names and predicted virulence according to the main associated biological characteristics, and references in Supplementary Table 5A (Excel file 1). [d] References: Kawai 2006[13]; Candan & Aksöz, 2015[14]; Paczosa et al. 2016[15]. [e]weak identity of 65% with 100% of coverage. [f]99-100% of global identity. [g]*clbS* gene absent in all strains. [h]we did not detect mutations in *kpnEFGH* genes. AMR, antimicrobial resistance; CG, clonal group; LPS, lipopolysaccharide; MLST, multilocus sequence typing; NE, not evaluable. Case number in order of strain detection.

Supplementary Table 6. Characteristics of plasmid structures in clinical ST437 polymyxin-carbapenem-resistant *K. pneumoniae* samples, according to Plasmidfinder[16] and Platon[17].

**CCBH Strains/Case #**		**17440^a/1**	**17428^a/2**	**19867/6**	**19771/8**
**MLST**		ST437	ST437	ST437	ST437
**Plasmid contig, #**		13	15	22	10
**Plasmid hit, #**		8	11	19	7
**Reference plasmid name (original host)**	**Accession #**	**Contig Length (kb), Alignment Start, Alignment End**			
pKPN-3967	NZ_CP026186.1	14.7, c-start=399, c-end=14408	14.7, c-start=299, c-end=14408	14.7, c-start=299, c-end=14408	14.7, c-start=299, c-end=14408
pAR-0427-1 (Escherichia coli O157)	NZ_CP044149.1	2.4, c-start=299, c-end=2409	1.9, c-start=1, c-end=1883	2.1, c-start=1, c-end=2112	1.9, c-start=1, c-end=1883
p1502320-3	NZ_CP031580.1	21.0, c-start=297, c-end=20659	21.0, c-start=297, c-end=20659	21.0, c-start=297, c-end=20659	21.0, c-start=297, c-end=20659
pKp145/11a	NZ_KX154765.1	42.0, c-start=1081, c-end=41991	42.0, c-start=1081, c-end=41991	7.2, c-start=1, c-end=7248	
p51015_NDM_1	NZ_CP050380.1	14.6, c-start=298, c-end=12419	14.6, c-start=298, c-end=12419	14.2, c-start=298, c-end=12419	
tig00000137 (*Proteus mirabilis)*	NZ_CP021551.1	9.0, c-start=1, c-end=8957	10.6, c-start=1, c-end=8974	9.0, c-start=1, c-end=8972	
pKPN528-2	NZ_CP020855.1		2.1, c-start=1, c-end=2104	2.1, c-start=1, c-end=2104	2.1, c-start=1, c-end=2104
pD120-1_296kb (*Klebsiella quasipneumoniae*)	NZ_CP034681.1		29.2, c-start=299, c-end=28878	29.2, c-start=299, c-end=28878	29.2, c-start=300, c-end=28879
pK66-45-1	NZ_CP020902.1		8.3, c-start=299, c-end=7981	8.3, c-start=300, c-end=7980	8.3, c-start=300, c-end=7982
Unnamed1 (*E. coli*)	NZ_CP042868.1		3.9, c-start=1, c-end=3847	3.8, c-start=1, c-end=3847	3.9, c-start=1, c-end=3847
pKp314/11a	NZ_KX276209.1	9.2			

pKPN528-1	NZ_CP020854.1	36.8			
pEC517_KPC (*E. coli*)	NZ_CP018963.1		9.1		
pEC656_KPC *(E. coli*)	NZ_CP018977.1			9.0	
pKPHS1	NC_016838.1			1.1	
p6234-178.193kb	NZ_CP010391.1			30.0	
pC9_003 (*Klebsiella aerogenes*)	NZ_CP042533.1			7.0	
Unnamed1	NZ_CP041935.1			30.7	
Unnamed1	NZ_CP018884.1			*8.9*	
tig00000727	NZ_CP021699.1			29.8	
pIMP69_000369 (*Providencia* sp. WCHPHu000369) /	NZ_CP031122.1			31.0	
pKPHS3 /	NC_016839.1			3.5	
**Replicon type, #**		6	6	7	5
IncFIB(pNDM-Mar)_1__JN420336					
IncHI1B(pNDM-MAR)_1__JN420336					
IncFIB(pKPHS1)					
Col440I					
Col(pHAD28)					

IncN	
IncA/C2	

[a] Index strains. Case number in order of strains detection. AMR, antimicrobial resistance; MLST, multilocus sequence typing.

Supplementary Table 7. Characteristics of plasmid structures in clinical ST11 polymyxin-carbapenem-resistant *K. pneumoniae* samples, according to Plasmidfinder[16] and Platon[17].

**CCBH Strains/Case #**		**17724/3**	**19868/7**
**MLST**		ST11	ST11
**Plasmid contig, #**		8	15
**Plasmid hit, #**		5	12
**Reference plasmid name (original host)**	**Accession #**	**Contig Length (kb), Alignment Start, Alignment End**	
pLH94-3	NZ_CP035205.1	60.0, c-start=5027, c-end=57749	58.0, c-start=5337, c-end=57647
pSF-173-1 (*E. coli*)	NZ_CP012632.1	11.8, c-start=300, c-end=11776	11.8, c-start=299, c-end=11774
p675SK2_B (*E. coli*)	NZ_CP027703.1	3.7, c-start=1, c-end=3323	3.7, c-start=1, c-end=3324
Unnamed1 (*Klebsiella aerogenes*)	NZ_CP031757.1	9.6, c-start=1, c-end=9232	9.6, c-start=1, c-end=9231
Unnamed1	NZ_CP028929.1	4.7	
p50595_IncFII	NZ_CP050373.1		21.5
pDA33141-21	NZ_CP029588.1		57.2
p6234-198.371kb	NZ_CP010390.1		4.5
pNDM5_020046	NZ_CP028781.1		*1.6*
pK66-45-2	NZ_CP020903.1		1.6
p1_020143	NZ_CP028543.1		22.8
pKP2_3	NZ_CP041949.1		33.8

pTetD_040074	NZ_CP029387.1		6.5
**Replicon type, #**		*5*	4
IncFIB(K)_1_Kpn3			
IncFII_1_pKP91			
Col440I			
Col(pHAD28)			
IncFII(pCRY)_1_pCRY			

Case number in order of strain detection. AMR, antimicrobial resistance; MLST, multilocus sequence typing

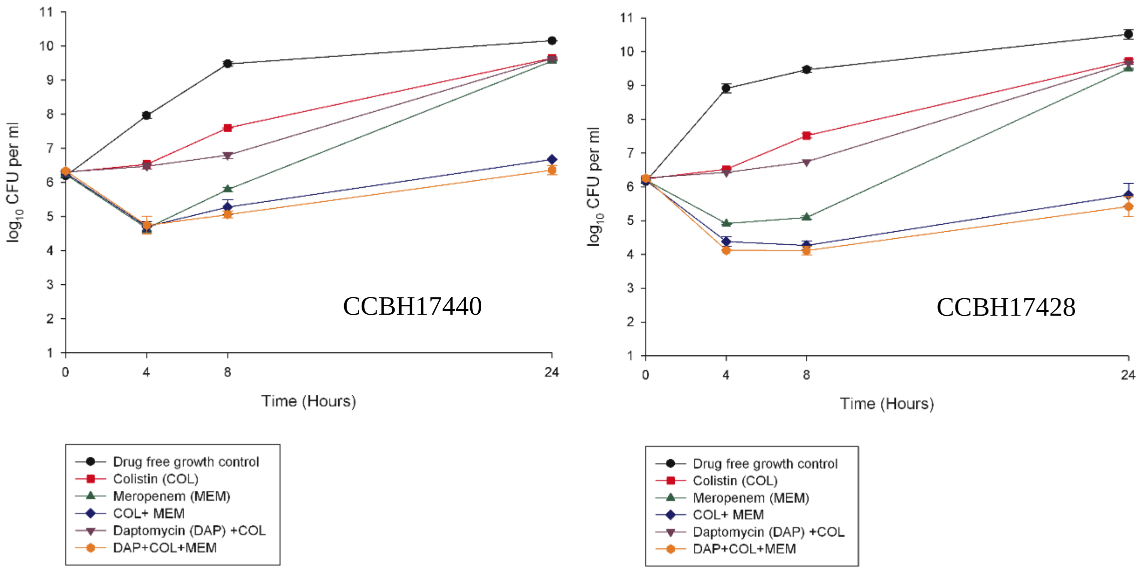

**Strain/case #**	**COL Alone**	**COL (+MEM)**	**MEM alone**	**MEM (+COL)**
CCBH17440/1	32 mg/L	16 mg/L	> 64 mg/L	64 mg/L
CCBH17428/2	64 mg/L	16 mg/L	> 64 mg/L	64 mg/L

Supplementary Figure 2. In vitro activity of colistin (COL) and meropenem (MEM) alone, and in combination of COL with MEM, daptomycin (DAP) and both against index ST437 polymyxin-carbapenem-resistant *K. pneumoniae* strains (CCBH17440, CCBH17428) by time kill curve method. MICs of MEM and COL alone and in combination. MEM concentration of 49 mg/L was used for combination MICs in presence of 0.5 x COL MIC.

References

1 CLSI. in *Twentieteh Fifth Informational Supplement. CLSI Document M100-S25* (ed Clinical Laboratory Standards Institute) (Wayne, PA, 2015).

2 Testing, T. E. C. o. A. S. *Breakpoint Tables for Interpretation of MICs and zone diameters.* , <http://www.eucast.org.> (2016).

3 Lam, M. M. C., Wick, R. R., Wyres, K. L. & Holt, K. E. Genomic surveillance framework and global population structure for Klebsiella pneumoniae. *bioRxiv*, 2020.2012.2014.422303, doi:10.1101/2020.12.14.422303 (2020).

4 Møller, T. S. B. *et al.* Relation between tetR and tetA expression in tetracycline resistant Escherichia coli. *BMC Microbiology* **16**, 39, doi:10.1186/s12866-016-0649-z (2016).

5 Wang, W. *et al.* High-level tetracycline resistance mediated by efflux pumps Tet(A) and Tet(A)-1 with two start codons. *J Med Microbiol* **63**, 1454-1459, doi:10.1099/jmm.0.078063-0 (2014).

6 Ahn, C., Yoon, S. S., Yong, T. S., Jeong, S. H. & Lee, K. The Resistance Mechanism and Clonal Distribution of Tigecycline-Nonsusceptible Klebsiella pneumoniae Isolates in Korea. *Yonsei Med J* **57**, 641-646, doi:10.3349/ymj.2016.57.3.641 (2016).

7 Chiu, S. K. *et al.* Roles of ramR and tet(A) Mutations in Conferring Tigecycline Resistance in Carbapenem-Resistant Klebsiella pneumoniae Clinical Isolates. *Antimicrob Agents Chemother* **61**, doi:10.1128/aac.00391-17 (2017).

8 Veleba, M., Higgins, P. G., Gonzalez, G., Seifert, H. & Schneiders, T. Characterization of RarA, a novel AraC family multidrug resistance regulator in Klebsiella pneumoniae. *Antimicrob Agents Chemother* **56**, 4450-4458, doi:10.1128/AAC.00456-12 (2012).

9 Veleba, M. & Schneiders, T. Tigecycline resistance can occur independently of the ramA gene in Klebsiella pneumoniae. *Antimicrobial agents and chemotherapy* **56**, 4466-4467, doi:10.1128/AAC.06224-11 (2012).

10 Markley, J. L. & Wencewicz, T. A. Tetracycline-Inactivating Enzymes. *Front Microbiol* **9**, 1058, doi:10.3389/fmicb.2018.01058 (2018).

11 Zhang, Q., Lin, L., Pan, Y. & Chen, J. Characterization of Tigecycline-Heteroresistant Klebsiella pneumoniae Clinical Isolates From a Chinese Tertiary Care Teaching Hospital. *Front Microbiol* **12**, 671153, doi:10.3389/fmicb.2021.671153 (2021).

12 Yang, X., Ye, L., Chan, E. W., Zhang, R. & Chen, S. Characterization of an IncFIB/IncHI1B Plasmid Encoding Efflux Pump TMexCD1-TOprJ1 in a Clinical Tigecycline- and Carbapenem-Resistant Klebsiella pneumoniae Strain. *Antimicrob Agents Chemother* **65**, doi:10.1128/AAC.02340-20 (2021).

13 Kawai, T. Hypermucoviscosity: an extremely sticky phenotype of Klebsiella pneumoniae associated with emerging destructive tissue abscess syndrome. *Clin Infect Dis* **42**, 1359-1361, doi:10.1086/503429 (2006).

14 Candan, E. D. & Aksoz, N. Klebsiella pneumoniae: characteristics of carbapenem resistance and virulence factors. *Acta Biochim Pol* **62**, 867-874, doi:10.18388/abp.2015_1148 (2015).

15 Paczosa, M. K. & Mecsas, J. Klebsiella pneumoniae: Going on the Offense with a Strong Defense. *Microbiol Mol Biol Rev* **80**, 629-661, doi:10.1128/MMBR.00078-15 (2016).

16 Carattoli, A. *et al.* In silico detection and typing of plasmids using PlasmidFinder and plasmid multilocus sequence typing. *Antimicrob Agents Chemother* **58**, 3895-3903, doi:10.1128/AAC.02412-14 (2014).

17 Schwengers, O. *et al.* Platon: identification and characterization of bacterial plasmid contigs in short-read draft assemblies exploiting protein sequence-based replicon distribution scores. *Microb Genom* **6**, doi:10.1099/mgen.0.000398 (2020).
